# On Consensus-Based Distributed Blind Calibration of Sensor Networks

**DOI:** 10.3390/s18114027

**Published:** 2018-11-19

**Authors:** Miloš S. Stanković, Srdjan S. Stanković, Karl Henrik Johansson, Marko Beko, Luis M. Camarinha-Matos

**Affiliations:** 1 Innovation Center, School of Electrical Engineering, University of Belgrade, 11120 Belgrade, Serbia; 2 Vlatacom Institute, 11070 Belgrade, Serbia; stankovic@etf.rs; 3 School of Technical Sciences, Singidunum University, 11000 Belgrade, Serbia; 4 School of Electrical Engineering, University of Belgrade, 11120 Belgrade, Serbia; 5 ACCESS Linnaeus Center, School of Electrical Engineering, KTH Royal Institute of Technology, SE-100 44 Stockholm, Sweden; kallej@kth.se; 6 COPELABS, Universidade Lusófona de Humanidades e Tecnologias, Campo Grande 376, 1749-024 Lisboa, Portugal; mbeko@uninova.pt; 7 CTS/UNINOVA , Monte de Caparica, 2829-516 Caparica, Portugal; cam@uninova.pt; 8 Faculty of Sciences and Technology, NOVA University of Lisbon, 2825-149 Caparica, Portugal

**Keywords:** blind calibration, macro calibration, distributed estimation, sensor networks, consensus, synchronization, stochastic approximation

## Abstract

This paper deals with recently proposed algorithms for real-time distributed blind macro-calibration of sensor networks based on consensus (synchronization). The algorithms are completely decentralized and do not require a fusion center. The goal is to consolidate all of the existing results on the subject, present them in a unified way, and provide additional important analysis of theoretical and practical issues that one can encounter when designing and applying the methodology. We first present the basic algorithm which estimates local calibration parameters by enforcing asymptotic consensus, in the mean-square sense and with probability one (w.p.1), on calibrated sensor gains and calibrated sensor offsets. For the more realistic case in which additive measurement noise, communication dropouts and additive communication noise are present, two algorithm modifications are discussed: one that uses a simple compensation term, and a more robust one based on an instrumental variable. The modified algorithms also achieve asymptotic agreement for calibrated sensor gains and offsets, in the mean-square sense and w.p.1. The convergence rate can be determined in terms of an upper bound on the mean-square error. The case when the communications between nodes is completely asynchronous, which is of substantial importance for real-world applications, is also presented. Suggestions for design of a priori adjustable weights are given. We also present the results for the case in which the underlying sensor network has a subset of (precalibrated) reference sensors with fixed calibration parameters. Wide applicability and efficacy of these algorithms are illustrated on several simulation examples. Finally, important open questions and future research directions are discussed.

## 1. Introduction

Recently emerged technologies dealing with networked systems, such as the Internet of Things (IoT), Networked Cyber-Physical Systems (CPS), and Sensor Networks (SN), still have many conceptual and practical challenges intriguing to both researchers and practitioners [1,2,3,4,5,6,7,8,9]. New classes of problems in this area continuously arise, driven by many new real-world applications. Particularly in the case of SNs, application examples include environment monitoring, wildfires detection, shop-floor manufacturing, smart cities, etc. One of the most important challenges, limiting the performance, robustness and time-to-market of these new technologies, is sensor calibration. Micro-calibration can be performed only in relatively small SNs where every sensor is individually calibrated in a controlled environment. Typical SNs are of large scale, functioning in dynamic and partially unobservable environments, thus demanding new methods and algorithms for efficient calibration. The idea of macro-calibration is to calibrate the entire SN based on the total system response, so that there is no requirement to individually calibrate every sensor node. The typical approach is to formulate the calibration problem as a parameter estimation problem (e.g., [10,11]). Of significant interest are methods for automatic calibration of SNs which successfully perform even if there are no reference signals/sensors, or other sources of groundthruth information about the measured process. In these situations, the goal of the calibration is to achieve homogeneous behavior of all the nodes, possibly enforcing dominant influence of sensors that are a priori known to provide sufficently good (calibrated) measurements. These types of calibration problems are known as the blind calibration problems (e.g., [12]). Furthermore, in many applications of SNs, it is of essential importance that the network functions in a completely decentralized fashion, preforming calibration in real-time, without the requirement for any kind of centralized information fusion. Hence, completely distributed and decentralized real-time calibration algorithms are of paramount importance.

In this paper, we study recently proposed algorithms which possess all the mentioned desirable properties: they deal with blind macro-calibration of SNs based on completely decentralized, real-time and recursive estimation of the parameters of linear calibration functions [13,14,15,16,17]. Another advantageous property of these algorithms is that it is assumed that the underlying SN have directed communication links between neighboring nodes. A basic algorithm is developed by using a distributed optimization problem setup, constructing a distributed gradient recursive scheme, with the local objectives formulated as weighted sums of mean-square differences between the corrected sensor readings of neighboring nodes. A direct consequence of this problem setup is that the algorithm can be studied as a generalized consensus scheme, to which the existing convergence results of standard consensus schemes are not applicable (e.g., [18]). However, by using techniques based on the stability of diagonally quasi-dominant dynamical systems [13,19,20,21] it is possible to prove asymptotic convergence of calibrated sensor outputs to consensus, in the mean-square sense and with probability one (w.p.1). The basic algorithm can be extended by assuming the presence of several factors which are of essential importance for practical applicability of the proposed method: (1) additive communication noise, (2) communication dropouts, (3) additive measurement noise, and (4) asynchronous communication.

Two possible modifications of the basic algorithm are presented for solving the problems posed in the cases (1)–(3) [16,17]. The first is based on the assumption that the noise variance is known a priori, which is used to design an appropriate compensation term [17]. The second modification is more robust and is based on an instrumental variable usage [16]. In both cases, the attainment of the asymptotic consensus in the mean-square sense and w.p.1 is guaranteed. In the case of completely asynchronous communication scenario, which is particularly important, we show how the algorithm can be implemented assuming a broadcast gossip communication scheme, which does not require clock synchronization among the agents, or any type of centralized information or coordination [14].

Another practically important situation arises when there are multiple nodes in the network that do not update (correct) their calibration parameters, but they still participate in the described distributed macro-calibration process. In this case, these nodes are called reference nodes since their only role is to provide reference information based on which other nodes should calibrate themselves. For example, this situation may arise in practice when a set of uncalibrated sensors is added to an already calibrated SN. In the case of more than one reference node, the corrected gains and offsets of the non-reference nodes, in general, do not converge to consensus, but to different points which depend on the information dictated by the reference sensors and the network properties [14]. In the case of only one reference sensor, the corrected gains and offsets of the rest of the sensors converge to the same point imposed by the reference sensor.

Finally, an analysis is given which clarifies the influence of initially selected weights corresponding to particular nodes in the presented calibration parameters estimation recursions. Guidelines are formulated on how these weights should be chosen so that given requirements are satisfied. General discussion of the described results is provided from both theoretical and practical points of view, based on which several future research directions are proposed.

The outline of the rest of the paper is as follows. The following section briefly discusses related work. In Section 3 we introduce the distributed blind macro-calibration problem and derive the basic algorithm for the noiseless case. Section 4 is devoted to the presentation of the convergence properties of the base algorithm. In Section 5 certain assumptions about the measured signals, communication errors, and communication protocol are relaxed, and the appropriate algorithm modifications are introduced, together with their convergence properties. In Section 6 a discussion on the convergence rate, the case of presence of reference sensors with fixed characteristics, and some design guidelines are presented. In Section 7 we present illustrative simulation results. Finally, Section 8 presents some conclusions and future research directions.

## 2. Related Work

Macro-calibration is based on the idea of calibrating the whole SN based on the responses of all the nodes. The most frequent approaches to this problem are based on parameter estimation techniques (e.g., [11]). If controlled stimuli are not available the problem is usually referred to as blind calibration of SNs. In general, it is a difficult problem, which has certain similarities with more general problems of blind estimation, equalization, and deconvolution (e.g., [22,23,24] and references therein).

Most of the proposed appraches to blind calibration in the existing literature are centralized and non-recursive [12,25,26,27,28,29,30,31,32,33,34,35,36,37]. Within this class of methods, in refs. [12,25] a blind calibration algorithm based on signal subspace projection was analyzed assuming restrictive signal and sensor properties. In ref. [26] the method was improved from the point of view of robustness to subspace uncertainties. In ref. [27] the authors proposed to use sparsity and convex optimization for blind estimation of calibration gains. In ref. [28] an approach to blind sensor calibration is adopted based on centralized consistency maximization at the network level assuming very dense deployment and only pairwise inter-node communications. In ref. [29] a moments-based centralized blind calibration is proposed for mobile SNs, exploiting multiple measurements of the same signal of mobile nodes, assuming that the measured signal does not change in time. In ref. [30], the authors proposed a method which can manage situations in which density requirements are not met. Interesting centralized approaches to blind drift calibration proposed in refs. [31,32,33], which also work when the density requirement is not met, are based on non-restrictive modeling of the assumed underlying signal subspace, with drift estimation using Kalman filter [31], sparse Bayesian learning [32], or deep learning [33]. The approach in ref. [34] also does not rely on stringent assumptions about signal subspace, but assume first-order auto-regressive signal process model. The authors of [35] introduce linear algebraic model of calibration relationships in a SN with centralized architecture to improve the simple mean calibration scheme, assuming sufficiently dense deployment. Another centralized approach to mobile sensors calibration is proposed in ref. [36] and is based on using a nonnegative matrix factorization. Some of the density assumptions introduced in this work were relaxed in ref. [37]. In ref. [38] the blind calibration problem was treated in a context of sparse sensing, using a message passing algorithm, assuming constant measured signal. The method proposed in ref. [39], based on geospatial estimation and Kalman filter, works if the sensors are calibrated at the beginning of the operation after deployment, and then may start to drift.

The problem of distributed blind macro-calibration may have certain similarities with the clock synchronization approaches based on local data processing and communications with neighbors [40,41,42,43,44,45,46,47]. However, these approaches cannot be directly mapped to the calibration problem treated in this paper.

Finally, certain extended consensus algorithms have been applied to SN calibration problems, but in different settings than the one treated in this paper [48,49,50,51]. An approach to blind calibration of sensor gains only, based on distributed gossip-based Expectation-Maximization iterations was proposed in ref. [52], assuming that the measured signal is constant. Another distributed approach was proposed in ref. [53], which explicitly uses a state-space model of the underlying process, and a message exchange protocol for offset compensation. The proposed scheme was formulated without proof of convergence. This paper is focused on the algorithms proposed recently in refs. [13,14,15,16,17] representing completely distributed and decentralized blind macro-calibration algorithms with rigorous proofs of convergences for both corrected sensor gains and offsets, with satisfactory performance under diverse deteriorating conditions which may typically appear in practical applications.

## 3. Problem Definition and the Basic Algorithm

Assume that the SN to be calibrated consists of *n* nodes/sensors. In the base setup, it is assumed that each sensor is measuring the same signal x(t) in discrete-time instants t=…,−1,0,1,…; this signal can be considered as a realization of a stochastic process {x(t)}. Note that we have implicitly assumed that the sensor nodes are functioning synchronously, since all the sensor nodes perform measurements in the same time instances *t*. We will relax this assumption in Section 5.3. The output (measurement) of the *i*-th node can be written as

(1) $$y_{i}\left(t\right)=\alpha_{i}x\left(t\right)+\beta_{i},$$

where $\alpha_{i}$ is the unknown gain, and $\beta_{i}$ the unknown offset of sensor *i*. Note that, in this problem setup, it is assumed that $\alpha_{i}$ and $\beta_{i}$ are unknown constants and not the random variables.

Calibration of a sensor is performed by applying an affine calibration function to the raw readings (1) which results in the following calibrated sensor output

(2) $$z_{i}\left(t\right)=a_{i}y_{i}\left(t\right)+b_{i}=g_{i}x\left(t\right)+f_{i},$$

where $a_{i}$ and $b_{i}$ are the calibration parameters to be obtained, $g_{i}=a_{i}\alpha_{i}$ is the corrected gain and $f_{i}=a_{i}\beta_{i}+b_{i}$ the corrected offset. The calibration objective is, ideally, to find parameters $a_{i}$ and $b_{i}$ for which $g_{i}$ is equal to one and $f_{i}$ equal to zero. In general, if we assume that there are no sensors which give perfect readings $z_{i}\left(t\right)=x\left(t\right)$ and that the signal x(t) is unknown and cannot be obtained or measured by some other means, this objective is impossible to achieve. Hence, in our decentralized real-time blind macro-calibration problem setup, this ideal objective must be alleviated: we require that the calibration process asymptotically achieves equal calibrated outputs $z_{i}\left(t\right)$ for all the nodes i=1,…,n. To approach as close as possible to the ideal goal of achieving $g_{i}=1$ and $f_{i}=0$, we could use certain a priori knowledge about the underlying SN, and try to adjust the algorithm, such that, loosely speaking, the “good” sensors (e.g., precalibrated or higher-quality sensors) correct, using the consensus strategy, the response of the rest of the sensors. For example, if, in a given SN, there is an apriori given perfectly calibrated reference sensor, the ideal asymptotic calibration ($g_{i}=1$ and $f_{i}=0$) of the rest of the sensor nodes will be achieved if the consensus goal is achieved.

It is assumed that the underlying SN have a predefined communication topology, defining possible inter-sensor communications, represented by a directed graph G=(N,E), where N is the set of nodes (sensors) and E the set of communication links (arcs). Define the adjacency matrix $A=[a_{ij}]$, i,j=1,…,n, where $a_{ij}=1$ if the *j*-th node is able to send messages to the *i*-th sensor, and $a_{ij}=0$ otherwise. Let $N_{i}$ be the set of in-neighboring nodes (or just neighbors) of the *i*-th node, i.e., the nodes *j* for which $a_{ij}=1$. Similarly, let $N_{i}^{\mathrm{out}}$ be the set of out-neighboring nodes of the *i*-th node, i.e., the nodes *j* for which $a_{ji}=1$.

Let us now derive the basic calibration algorithm. The idea is to start with local criteria for each node, whose local minimization would lead to a network-level consensus on the corrected sensor outputs:

(3) $$\begin{array}{c} J_{i}=\sum_{j\in N_{i}}\gamma_{ij}E\left\{{\left(z_{j}\left(t\right)-z_{i}\left(t\right)\right)}^{2}\right\}, \end{array}$$

i=1,…,n, where $\gamma_{ij}$ are nonnegative scalar weights whose influence on the properties of the algorithm will be discussed later. Denoting $\theta_{i}={\left[a_{i}\;\;b_{i}\right]}^{T}$, the following expression is obtained for the gradient of (3):

(4) $${\mathrm{grad}}_{\theta_{i}}J_{i}=\sum_{j\in N_{i}}\gamma_{ij}E\left\{\left(z_{j}\left(t\right)-z_{i}\left(t\right)\right)\left[\begin{array}{c} y_{i}\left(t\right)\\ 1 \end{array}\right]\right\}.$$

From (4) we obtain the following stochastic gradient recursion for estimating $\theta_{i}^{*}$ minimizing (3):

(5) $${\hat{\theta }}_{i}\left(t+1\right)={\hat{\theta }}_{i}\left(t\right)+\delta_{i}\left(t\right)\sum_{j\in N_{i}}\gamma_{ij}\epsilon_{ij}\left(t\right)\left[\begin{array}{c} y_{i}\left(t\right)\\ 1 \end{array}\right],$$

where ${\hat{\theta }}_{i}\left(t\right)={\left[{\hat{a}}_{i}\left(t\right)\;\;{\hat{b}}_{i}\left(t\right)\right]}^{T}$, $\epsilon_{ij}\left(t\right)={\hat{z}}_{j}\left(t\right)-{\hat{z}}_{i}\left(t\right)$, ${\hat{z}}_{i}\left(t\right)={\hat{a}}_{i}\left(t\right)y_{i}\left(t\right)+{\hat{b}}_{i}\left(t\right)$, and $\delta_{i}\left(t\right)>0$ is a time-varying gain whose influence on the convergence properties of the algorithm will be discussed later. The initial conditions are assumed to be ${\hat{\theta }}_{i}\left(0\right)={\left[1\;\;0\right]}^{T}$, i=1,…,n. We expect that the set of recursions (5) asymptotically achieve that all the local estimates of corrected gains ${\hat{g}}_{i}\left(t\right)={\hat{a}}_{i}\left(t\right)\alpha_{i}$ and corrected offsets ${\hat{f}}_{i}\left(t\right)={\hat{a}}_{i}\left(t\right)\beta_{i}+{\hat{b}}_{i}\left(t\right)$ converge to the same values $\bar{g}$ and $\bar{f}$, respectively; this implies that the corrected sensor outputs of all the nodes are also equal ${\hat{z}}_{j}\left(t\right)={\hat{z}}_{i}\left(t\right)$, i,j=1,…,n.

**Figure 1:**
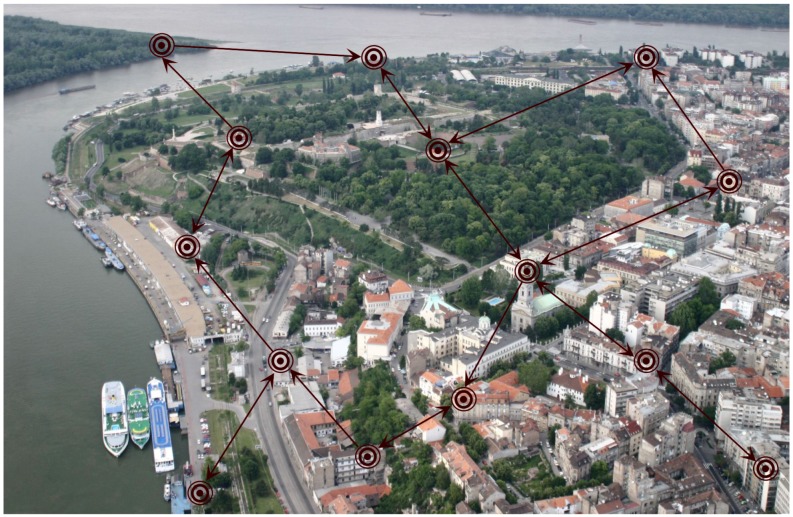
An example sensor network used in smart-city applications with decentralized communication topology. The inter-node communication is performed according to the depicted directed graph. The introduced distributed calibration algorithm achieves asymptotic calibration of all the sensor nodes in the network without using any type of fusion center.

In Figure 1 an illustrating smart-city example sensor network is depicted. Completely decentralized network architecture is assumed, i.e., the nodes communicate according to the directed communication graph which is represented in the figure using arcs. The communication graph will typically depend on the mutual node distances, transmission power of individual nodes, channel conditions, presence of obstacles, etc. Each node in the network is equipped with the same type of sensor which measures certain physical quantity (e.g., certain atmospheric condition or air quality indicator). At each time instant *t*, a node *i* performs local reading of the raw sensor output $y_{i}\left(t\right)$, calculation of the corrected sensor output ${\hat{z}}_{i}\left(t\right)$ according to (2) using current local estimates of the calibration parameters ${\hat{a}}_{i}\left(t\right)$ and ${\hat{b}}_{i}\left(t\right)$, transmission of the corrected value ${\hat{z}}_{i}\left(t\right)$ to the out-neighbors $N_{i}^{\mathrm{out}}$, reception of the values ${\hat{z}}_{j}\left(t\right)$ from the in-neighbors $j\in N_{i}$, and calculation of the updated estimates of the local calibration parameters ${\hat{a}}_{i}\left(t+1\right)$ and ${\hat{b}}_{i}\left(t+1\right)$ using (5). In the initial presentation we will assume that, at each iteration of the algorithm (5), local sensor measurement $y_{i}\left(t\right)$ and the current messages of the neighboring nodes’ corrected outputs ${\hat{z}}_{j}\left(t\right)$ are available at node *i*. Possible communication dropouts and/or faulty/noisy sensor readings will be treated later. Local computational cost for each agent is minor since only two parameters are being estimated. Communication complexity depends on the number of neighboring agents, which is small in typical SNs with decentralized architecture.

For the sake of compact notations, suitable for convergence analysis of the derived algorithm, let us introduce

(6) $${\hat{\phi }}_{i}\left(t\right)=\left[\begin{array}{c} {\hat{g}}_{i}\left(t\right)\\ {\hat{f}}_{i}\left(t\right) \end{array}\right]=\left[\begin{array}{cc} \alpha_{i} & 0\\ \beta_{i} & 1 \end{array}\right]{\hat{\theta }}_{i}\left(t\right),$$

and

(7) $$\epsilon_{ij}\left(t\right)=\left[\begin{array}{cc} x(t) & 1 \end{array}\right]\left({\hat{\phi }}_{j}\left(t\right)-{\hat{\phi }}_{i}\left(t\right)\right),$$

so that (5) becomes

(8) $${\hat{\phi }}_{i}\left(t+1\right)={\hat{\phi }}_{i}\left(t\right)+\delta_{i}\left(t\right)\sum_{j\in N_{i}}\gamma_{ij}\Omega_{i}\left(t\right)\left({\hat{\phi }}_{j}\left(t\right)-{\hat{\phi }}_{i}\left(t\right)\right),$$

where

(9) $$\begin{array}{cc} \Omega_{i}\left(t\right)= & \left[\begin{array}{cc} \alpha_{i}y_{i}\left(t\right)x\left(t\right) & \alpha_{i}y_{i}\left(t\right)\\ \beta_{i}y_{i}\left(t\right)]x\left(t\right) & 1+\beta_{i}y_{i}\left(t\right) \end{array}\right]\\ = & \left[\begin{array}{cc} \alpha_{i}\beta_{i}x\left(t\right)+\alpha_{i}^{2}x{\left(t\right)}^{2} & \alpha_{i}\beta_{i}+\alpha_{i}^{2}x\left(t\right)\\ \left(1+\beta_{i}^{2}\right)x\left(t\right)+\alpha_{i}\beta_{i}x{\left(t\right)}^{2} & 1+\beta_{i}^{2}+\alpha_{i}\beta_{i}x\left(t\right) \end{array}\right], \end{array}$$

with the initial conditions ${\hat{\rho }}_{i}\left(0\right)={\left[\alpha_{i}\;\;\beta_{i}\right]}^{T},\;i=1,\ldots ,n$. Therefore, the following compact form for the recursions (8) is obtained

(10) $$\hat{\phi }\left(t+1\right)=\left[I+\left(\Delta \left(t\right)⊗I_{2}\right)B\left(t\right)\right]\hat{\phi }\left(t\right),$$

where ⊗ is the Kronecker product, *I* is the identity matrix of dimension 2n, $I_{2}$ is the dimension 2 identity matrix, $\hat{\phi }\left(t\right)={\left[{\hat{\phi }}_{1}{\left(t\right)}^{T}\cdots {\hat{\phi }}_{n}{\left(t\right)}^{T}\right]}^{T},\;\Delta \left(t\right)=\mathrm{diag}\{\delta_{1}\left(t\right),\ldots ,\delta_{n}\left(t\right)\}$, diag{…} denotes the corresponding block diagonal matrix,

$$B\left(t\right)=\Omega \left(t\right)\left(\Gamma ⊗I_{2}\right),$$

$$\Omega \left(t\right)=\mathrm{diag}\{\Omega_{1}\left(t\right),\ldots ,\Omega_{n}\left(t\right)\},$$

$$\Gamma =\left[\begin{array}{cccc} -\sum_{j,j\neq 1}\gamma_{1j} & \gamma_{12} & \cdots & \gamma_{1n}\\ \gamma_{21} & -\sum_{j,j\neq 2}\gamma_{2j} & \cdots & \gamma_{2n}\\ \ddots \\ \gamma_{n1} & \gamma_{n2} & \cdots & -\sum_{j,j\neq n}\gamma_{nj} \end{array}\right],$$

where $\gamma_{ij}=0$ when $j\notin N_{i}$, and the initial condition is $\hat{\phi }\left(0\right)={\left[{\hat{\phi }}_{1}{\left(0\right)}^{T}\cdots {\hat{\phi }}_{n}{\left(0\right)}^{T}\right]}^{T},$ according to (8). From the way in which we have constructed the vector $\hat{\phi }\left(t\right)$ we conclude that the asymptotic value of $\hat{\phi }\left(t\right)$ should be such that all of its odd components are equal, and all of its even components are equal.

In the next section, it will be shown that, under certain general assumptions, for any choice of the weights $\gamma_{ij}\geq 0$ for $j\in N_{i}$ (and $\gamma_{ij}=0$ when $j\notin N_{i}$) the algorithm achieves convergence to consensus. However, if the underlying calibration objective is to achieve absolute calibration of the sensors (i.e., $\bar{g}$ close to one and $\bar{f}$ close to zero), this can be done by trying to exploit sensors that are a priori known to have good characteristics. In a large SN, this can be achieved in two ways: (1) if the large number of sensors are “good” sensors, then $\gamma_{ij}$-s in all neighborhoods $N_{i}$ should be approximately the same; or (2) if there is a set of a priori chosen good sensors $j\in N^{f}\subset N$ the goal is to enforce their dominant influence to the rest of the nodes. There are two possibilities to achieve this: (a) to set high values of $\gamma_{ij}$ for all $j\in N^{f}$ and $i\in N_{j}^{\mathrm{out}}$; or (b) to set small values of $\gamma_{jk}$ for all $j\in N^{f}$, $k\in N_{j}$, k≠j (which prevents large changes of ${\hat{\phi }}_{j}\left(t\right)$). Section 6.3 deals with the guidelines on weights tuning, while Section 6.4 treats the case in which a set of reference sensors is kept with fixed calibration parameters.

## 4. Convergence Analysis

In this section we discuss the convergence properties of the calibration scheme presented in the previous section, where it has been assumed that both local sensor measurements and inter-node communications are perfect, i.e., possible communication errors and/or measurement errors are not present. We first analyze this basic scheme in order to focus on structural characteristics of the algorithm; the case of lossy SNs will be treated in the subsequent sections. In the basic setup, without presence of any unreliability, it is sufficient to assume that the step sizes $\delta_{i}\left(t\right)$ are constant:

(A1) $\delta_{i}\left(t\right)=\delta =\mathrm{const}$, for all i=1,…,n.

For clearer initial presentation of the convergence results, we now adopt a simplifying assumption:

(A2) {x(t)} is independent and identically distributed (i.i.d.) sequence, with $E\left\{x\left(t\right)\right\}=\bar{x}<\infty$ and $E\left\{x{\left(t\right)}^{2}\right\}=s^{2}<\infty$.

In practice, when the SNs are used to measure certain physical quantities, the assumption that {x(t)} is i.i.d. is almost never satisfied; hence it will be relaxed later.

Based on (A1) and (A2), the expectation of the parameter estimates $\bar{\phi }\left(t\right)=E\left\{\phi \left(t\right)\right\}$ satisfies the following recursion

(11) $$\bar{\phi }\left(t+1\right)=\left(I+\delta \bar{B}\right)\bar{\phi }\left(t\right),$$

where $\bar{\phi }\left(0\right)=\phi \left(0\right)$, $\bar{B}=\bar{\Omega }\left(\Gamma ⊗I_{2}\right)$ and $\bar{\Omega }=E\left\{\Omega \left(t\right)\right\}=\mathrm{diag}\left\{{\bar{\Omega }}_{1}\ldots {\bar{\Omega }}_{n}\right\}$, with

(12) $${\bar{\Omega }}_{i}=\left[\begin{array}{cc} \alpha_{i}\beta_{i}\bar{x}+\alpha_{i}^{2}s^{2} & \alpha_{i}\beta_{i}+\alpha_{i}^{2}\bar{x}\\ \left(1+\beta_{i}^{2}\right)\bar{x}+\alpha_{i}\beta_{i}s^{2} & 1+\beta_{i}^{2}+\alpha_{i}\beta_{i}\bar{x} \end{array}\right].$$

The following assumption, typical for consensus-based algorithms, is introduced:

(A3) Graph G has a spanning tree.

It implies that the matrix Γ has one zero eigenvalue and the rest eigenvalues with negative real parts, e.g., [54]. Hence, from the structure of matrix $\bar{B}$, we directly conclude that it has at least two zero eigenvalues. Its remaining eigenvalues can be characterized starting from the following assumption:

(A4) $s^{2}-{\bar{x}}^{2}=\mathrm{var}\left\{x\left(t\right)\right\}>0$.

This assumption guarantees that the estimation recursions are sufficiently excited by the signal x(t). Its important consequence is that $-{\bar{\Omega }}_{i}$ defined by (12) is Hurwitz, for all i=1,…,n. Indeed, using some simple algebra it can be derived that $-{\bar{\Omega }}_{i}$ is Hurwitz if and only if (iff)

(13) $$\alpha_{i}^{2}\left(s^{2}-{\bar{x}}^{2}\right)>0,\;\;\;2\alpha_{i}\beta_{i}\bar{x}+\alpha_{i}^{2}s^{2}+1+\beta_{i}^{2}>0.$$

Both inequalities hold iff (A4) holds. This greatly simplifies further derivations which depend on somewhat complicate expression (12) for the 2×2 diagonal blocks of the matrix $\bar{\Omega }$.

Because of the block structure of matrices $\bar{\Omega }$ and $\bar{B}$, the properties of the main recursion (11) cannot be analyzed using standard linear consensus methodologies (see, e.g., [18,54] and references therein). To cope with this problem, a methodology based on the concept of diagonal quasi-dominance of matrices decomposed into blocks has been used [13,17,19,20,21] to obtain the following important result characterizing all the eigenvalues of the matrix $\bar{B}$.

**Lemma** **1** ([13,17])**.** *Assume that the assumptions (A3) and (A4) hold. Then, matrix $\bar{B}$ in (11) has two zero eigenvalues and the rest eigenvalues have negative real parts.*

Observe that vectors $i_{1}={\left[\begin{array}{ccccccc} 1 & 0 & 1 & 0 & \ldots & 1 & 0 \end{array}\right]}^{T}\in R^{2n}$ and $i_{2}={\left[\begin{array}{ccccccc} 0 & 1 & 0 & 1 & \ldots & 0 & 1 \end{array}\right]}^{T}\in R^{2n}$, where R is the set of real numbers, are the right eigenvectors of $\bar{B}$ corresponding to the eigenvalue at the origin. Let $\rho_{1}$ and $\rho_{2}$ be the corresponding normalized left eigenvectors, satisfying $\left[\begin{array}{c} \rho_{1}\\ \rho_{2} \end{array}\right]\left[\begin{array}{cc} i_{1} & i_{2} \end{array}\right]=I_{2}$. The following lemma deals with a similarity transformation important for all the remaining derivations throughout the paper.

**Lemma** **2** ([13,17])**.** *Let $T=\left[\begin{array}{ccc} i_{1} & i_{2} & T_{2n\times (2n-2)} \end{array}\right]$, where $T_{2n\times (2n-2)}$ is an 2n×(2n−2) matrix, such that span$\left\{T_{2n\times (2n-2)}\}=\mathrm{span}\{\bar{B}\right\}$ (span{A} denotes a linear space spanned by the columns of matrix A). Then, T is nonsingular and*

(14) $$T^{-1}\bar{B}T=\left[\begin{array}{cc} 0_{2\times 2} & 0_{2\times (2n-2)}\\ 0_{(2n-2)\times 2} & {\bar{B}}^{*} \end{array}\right],$$

*where ${\bar{B}}^{*}$ is Hurwitz, and $0_{i\times j}$ denotes a i×j zero matrix.*

Notice that

(15) $$T^{-1}=\left[\begin{array}{c} \rho_{1}\\ \rho_{2}\\ S_{(2n-2)\times 2n} \end{array}\right],$$

where $S_{(2n-2)\times 2n}$ can be determined from the definition of *T*.

From the structure of the matrices in (11), it can be concluded that the transformation *T* from Lemma 2, when applied to the original matrix B(t), will produce a matrix which has the same structure as the transformed matrix given in Equation (14).

**Lemma** **3** ([13,17])**.** *For the matrix B(t) in (10) it holds that, for all t,*

(16) $$T^{-1}B\left(t\right)T=\left[\begin{array}{cc} 0_{2\times 2} & 0_{2\times (2n-2)}\\ 0_{(2n-2)\times 2} & B{\left(t\right)}^{*} \end{array}\right],$$

*where $B{\left(t\right)}^{*}$ is an (2n−2)×(2n−2) matrix and T is given in Lemma 2.*

The following convergence theorem can now be formulated.

**Theorem** **1** ([17])**.** *Assume that Assumptions (A1)–(A4) hold. Then there exists $\delta^{\prime }>0$ such that for all $\delta \leq \delta^{\prime }$ in (10)*

(17) $$\lim_{t\to \infty }\hat{\phi }\left(t\right)=\left(i_{1}\rho_{1}+i_{2}\rho_{2}\right)\hat{\phi }\left(0\right)$$

*in the mean square sense and w.p.1.*

Note here that the limit vector in (17) $\left(i_{1}\rho_{1}+i_{2}\rho_{2}\right)\hat{\phi }\left(0\right)$ have all the odd elements equal, and all the even elements equal, which means that the corrected gains of all the nodes converge to the same value, and the corrected offsets of all the nodes converge to the same value. It can be shown [13] that this value only depends on the unknown sensor parameters $\alpha_{i}$ and $\beta_{i}$, and the weights $\gamma_{ij}$ in $J_{i}$, i,j=1,…n. For given initial conditions in (5), $\rho_{1}\hat{\phi }\left(0\right)$ and $\rho_{2}\hat{\phi }\left(0\right)$ are in the form of weighted sums of $\alpha_{i}$ and $\beta_{i}$, 1,…,n, respectively. Assuming that the weights $\gamma_{ij}$ are the same for all the nodes, and that $\alpha_{i}$ have a distribution centered around one, and $\beta_{i}$ around zero, these weighted sums will be close to one and zero, respectively.

The value of $\delta^{\prime }>0$ in Theorem 1, which ensures convergence, may be restrictive. In practice, the choice of step size δ in (A1) should be based on the actual properties of the underlying SN; its value needs to be small enough to achieve convergence, but it should also be sufficiently large to achieve acceptable rate of convergence (as in the standard parameter estimation recursions [55]).

After clarifying the main structural properties of the algorithm, we now treat the more realistic case of correlated sequences {x(t)}. We replace (A2) with:

(A2’) The random process {x(t)} is weakly stationary, bounded w.p.1, and with bounded first and second moments, i.e., |x(t)|≤K<∞, $E\left\{x\left(t\right)\right\}=\bar{x}<\infty$, E{x(t−d)x(t)}=m(d)<∞ for all d∈{0,1,2,…} (E{·} is a sign of the mathematical expectation), $m\left(0\right)=s^{2}<\infty$. It also holds that

(18) $$\begin{array}{cc} \left(a\right) & \;\;\;\;\;|E\left\{x\left(t\right)|F_{t-\tau }\right\}-\bar{x}|=o\left(1\right),\;\;\left(w.p.1\right) \end{array}$$

(19) $$\begin{array}{cc} \left(b\right) & \;\;\;\;\;|E\{x\left(t-d\right)x\left(t\right)|F_{t-\tau }\}-m\left(d\right)|=o\left(1\right),\;\;\left(w.p.1\right) \end{array}$$

when τ→∞, for all d∈{0,1,2,…}, τ>d ($F_{t-\tau }$ denotes the minimal σ-algebra generated by {x(0),x(1),…,x(t−τ)}, and o(1) denotes a function that converges to zero when τ→∞).

Hence, (A2’) requires stationarity, boundedness, and imposes a mixing condition on the signal {x(t)}. The explicitly used time shift parameter *d* will be used later for introducing a new algorithm based on an instrumental variable, capable of dealing with possible measurement noise.

The following theorem examines the convergence of the algorithm (11) under assumption (A2’):

**Theorem** **2**  ([16])**.** *Assume that the assumptions (A1), (A2’), (A3) and (A4) hold. Then there exists $\delta^{\prime \prime }>0$ such that for all $\delta \leq \delta^{\prime \prime }$ in (10) $\lim_{t\to \infty }\hat{\phi }\left(t\right)=\left(i_{1}\rho_{1}+i_{2}\rho_{2}\right)\hat{\phi }\left(0\right)$ in the mean square sense and w.p.1.*

## 5. Extensions of the Basic Algorithm

In this section, we introduce several modifications and generalizations of the basic algorithm (5), so that it is possible to achieve distributed calibration under more challenging conditions, typically present in real-life SNs: communication dropouts, additive communication noise, measurement noise, and asynchronous communication. Convergence properties of the introduced modifications are presented in detail.

### 5.1. Communication Errors

In this subsection, we assume that inter-node communication errors can be manifested in two ways: (1) communication dropouts (outages) and (2) additive communication noise. Communication dropouts typically occur in SNs using digital communication; additive noise can, in this case, model quantization effects. For example, in the case of smart city sensor networks, depicted in Figure 1, the dropouts will happen relatively often because of the dynamic environment, where both physical obstacles and electronic interference can be persistent. In certain, less frequent practical situations, SNs can use analog communication (e.g., when certain types of energy harvesting are used [56]), when additive communication noise is dominant, and dropouts appear less frequently.

The communication errors are formally introduced using the following assumptions:

(A5) The weights $\gamma_{ij}$ in the algorithm (5) are now randomly time-varying, according to stochastic processes given by $\left\{\gamma_{ij}\left(t\right)\right\}=\left\{u_{ij}\left(t\right)\gamma_{ij}\right\}$, where $\left\{u_{ij}\left(t\right)\right\}$ are i.i.d. binary random sequences, such that $u_{ij}\left(t\right)=1$ with probability $p_{ij}$ ($p_{ij}>0$ when $j\in N_{i}$), and $u_{ij}\left(t\right)=0$ with probability $1-p_{ij}$.

(A6) Instead of receiving ${\hat{z}}_{j}\left(t\right)$ from the *j*-th node, the *i*-th node receives ${\hat{z}}_{j}\left(t\right)+\xi_{ij}\left(t\right)$, where $\left\{\xi_{ij}\left(t\right)\right\}$ is an i.i.d. random sequence with $E\{\xi_{ij}\left(t\right)\}=0$ and $E\left\{\xi_{ij}{\left(t\right)}^{2}\right\}={\left(\sigma_{ij}^{\xi }\right)}^{2}<\infty$.

(A7) Processes {x(t)}, $\left\{u_{ij}\left(t\right)\right\}$ and $\left\{\xi_{ij}\left(t\right)\right\}$ are mutually independent.

Based on the above assumptions, the communication dropout at any iteration *t*, when node *j* is sending to node *i*, will happen with probability $1-p_{ij}$, independently of the additive communication noise process $\left\{\xi_{ij}\left(t\right)\right\}$ and the measured signal {x(t)}.

Denoting

$$\nu_{i}\left(t\right)=\sum_{j\in N_{i}}\gamma_{ij}\left(t\right)\xi_{ij}\left(t\right)\left[\begin{array}{c} \alpha_{i}y_{i}\left(t\right)\\ 1+\beta_{i}y_{i}\left(t\right) \end{array}\right],$$

and $\nu \left(t\right)=\left[\begin{array}{ccc} \nu_{1}\left(t\right) & \ldots & \nu_{n}\left(t\right) \end{array}\right]$, one obtains from (10) that

(20) $$\hat{\phi }\left(t+1\right)=\left[I+\left(\Delta \left(t\right)⊗I_{2}\right)B^{\prime }\left(t\right)\right]\hat{\phi }\left(t\right)+\Delta \left(t\right)\nu \left(t\right),$$

where $B^{\prime }\left(t\right)=\Omega \left(t\right)\left(\Gamma \left(t\right)⊗I_{2}\right)$, and Γ(t) is obtained from Γ by applying (A5).

Convergence properties of the recursion (20), under the additional assumptions (A5)–(A7), can be derived starting from the results of the previous subsection. Due to the mutual independence of the random variables in $B^{\prime }\left(t\right)$, it can be concluded that $E\left\{B^{\prime }\left(t\right)\right\}={\bar{B}}^{\prime }=\bar{\Omega }\left(\bar{\Gamma }⊗I_{2}\right)$, where $\bar{\Gamma }=E\left\{\Gamma \left(t\right)\right\}$ is the same as Γ but with $\gamma_{ij}$ replaced by $\gamma_{ij}p_{ij}$. Also, it follows that ${\tilde{B}}^{\prime }\left(t\right)≐B^{\prime }\left(t\right)-{\bar{B}}^{\prime }$, is a martingale difference sequence (since $E\{{\tilde{B}}^{\prime }\left(t\right)|F_{t-1}\}=0$). Furthermore, it can be concluded that ${\bar{B}}^{\prime }=\bar{\Omega }\left(\bar{\Gamma }⊗I_{2}\right)$ has the same spectrum as $\bar{B}$ in (11): it has two zero eigenvalues and the rest eigenvalues are with negative real part.

Since the additive noise is now present in the recursions (20), (A1) needs to be replaced with the following assumption, typical in the stochastic approximation literature (e.g., [57]):

(A1’) $\delta_{i}\left(t\right)=\delta \left(t\right)>0$, $\sum_{t=0}^{\infty }\delta \left(t\right)=\infty$, $\sum_{t=0}^{\infty }\delta {\left(t\right)}^{2}<\infty$, i=1,…,n.

Intuitively, (A1’) introduces diminishing gains $\delta_{i}\left(t\right)$ which converge to zero slowly enough, so that the additive noise can be averaged out while asymptotic convergence to a consensus point is achieved (despite the presence of noise).

Therefore, we have

(21) $$\hat{\phi }\left(t+1\right)=\left(I+\delta \left(t\right){\bar{B}}^{\prime }\right)\hat{\phi }\left(t\right)+\delta \left(t\right){\tilde{B}}^{\prime }\left(t\right)\hat{\phi }\left(t\right)+\delta \left(t\right)\nu \left(t\right).$$

Similarly as in the noiseless case, let as introduce the similarity transformation

$$T^{\prime }=\left[\begin{array}{ccc} i_{1} & i_{2} & T_{2n\times (2n-2)}^{\prime } \end{array}\right],$$

where $T_{2n\times (2n-2)}^{\prime }$ is an 2n×(2n−2) matrix, such that $\mathrm{span}\left\{T_{2n\times (2n-2)}^{\prime }\right\}=\mathrm{span}\left\{{\bar{B}}^{\prime }\right\}$. Then, ${\left(T^{\prime }\right)}^{-1}=\left[\begin{array}{c} \rho_{1}^{\prime }\\ \rho_{2}^{\prime }\\ S_{(2n-2)\times 2n}^{\prime } \end{array}\right]$, where $\rho_{1}^{\prime }$ and $\rho_{2}^{\prime }$ are the left eigenvectors of ${\bar{B}}^{\prime }$ corresponding to the eigenvalue at the origin. By applying transformation $T^{\prime }$ to (21), and using stochastic Lyapunov stability arguments, along with the arguments typically used in analyzing stochastic approximation algorithms [13,17,58,59], the following theorem can be proved:

**Theorem** **3** ([13,17])**.** *Let Assumptions (A1’), (A2)–(A7) be satisfied. Then, $\hat{\phi }\left(t\right)$ generated by (21) converges to $i_{1}w_{1}+i_{2}w_{2}$ in the mean square sense and w.p.1, where $w_{1}$ and $w_{2}$ are scalar random variables satisfying $E\left\{w_{1}\right\}=\rho_{1}^{\prime }\hat{\phi }\left(0\right)$ and $E\left\{w_{2}\right\}=\rho_{2}^{\prime }\hat{\phi }\left(0\right)$.*

The theorem essentially states that, again, all the corrected drifts converge to the same point, and all the corrected offsets converge to the same point; however, because of the additive communication noise, these points are random and depend on the noise realization. The mean values of these possible convergence points depend on the sensor parameters $\alpha_{i}$ and $\beta_{i}$, the design parameters $\gamma_{ij}$, as well as on the dropout probabilities $p_{ij}$, i,j=1,…n.

### 5.2. Measurement Noise

In this subsection we, in addition to communication errors, assume that the signal x(t) is measured with additive measurement noise. This situation is of essential importance for practical applications since practically all the existing sensors contain certain measurement errors which are typically modeled using stochastic processes [3].

Formally, we model the additive noise stochastic process using the following assumption:

(A8) Instead of $y_{i}\left(t\right)$ given by (1), the sensor measurements are now contaminated by noise, and given by

$$y_{i}^{\eta }\left(t\right)=\alpha_{i}x\left(t\right)+\beta_{i}+\eta_{i}\left(t\right),$$

where $\left\{\eta_{i}\left(t\right)\right\}$, i=1,…n, are zero mean i.i.d. random sequences with $E\left\{\eta_{i}{\left(t\right)}^{2}\right\}={\left(\sigma_{i}^{\eta }\right)}^{2}$, independent of the measured signal x(t).

By replacing $y_{i}^{\eta }\left(t\right)$ instead of $y_{i}\left(t\right)$ in the base algorithm (5), one obtains the following “noisy” version of (8):

(22) $$\begin{array}{cc} {\hat{\phi }}_{i}\left(t+1\right)= & {\hat{\phi }}_{i}\left(t\right)+\delta_{i}\left(t\right)\sum_{j\in N_{i}}\gamma_{ij}\left\{\left[\Omega_{i}\left(t\right)+\Psi_{i}\left(t\right)\right]\left[{\hat{\phi }}_{j}\left(t\right)-{\hat{\phi }}_{i}\left(t\right)\right]+N_{ij}\left(t\right){\hat{\phi }}_{j}\left(t\right)-N_{ii}\left(t\right){\hat{\phi }}_{i}\left(t\right)\right\}, \end{array}$$

where $\Psi_{i}\left(t\right)=\eta_{i}\left(t\right)\left[\begin{array}{cc} \alpha_{i}x\left(t\right) & \alpha_{i}\\ \beta_{i}x\left(t\right) & \beta_{i} \end{array}\right],$ $N_{ij}\left(t\right)=\frac{\eta_{j}\left(t\right)}{\alpha_{j}}\left[\begin{array}{cc} \alpha_{i}y_{i}\left(t\right) & 0\\ \beta_{i}y_{i}\left(t\right) & 0 \end{array}\right]+\left[\begin{array}{cc} \frac{\eta_{j}\left(t\right)\eta_{i}\left(t\right)}{\alpha_{j}} & 0\\ 0 & 0 \end{array}\right]$ and $N_{ii}\left(t\right)=\frac{\eta_{i}\left(t\right)}{\alpha_{i}}\left[\begin{array}{cc} \alpha_{i}y_{i}\left(t\right) & 0\\ \beta_{i}y_{i}\left(t\right) & 0 \end{array}\right]+\left[\begin{array}{cc} \frac{\eta_{i}{\left(t\right)}^{2}}{\alpha_{i}} & 0\\ 0 & 0 \end{array}\right]$, assuming $\alpha_{i}\neq 0$, i=1,…,n. It is important to observe here that $E\{\Psi_{i}\left(t\right)\}=0$, $E\{N_{ij}\left(t\right)\}=0$; however $E\left\{N_{ii}\left(t\right)\right\}=\left[\begin{array}{cc} \frac{{\left(\sigma_{i}^{\eta }\right)}^{2}}{\alpha_{i}} & 0\\ 0 & 0 \end{array}\right]$.

Assuming again that the step sizes $\delta_{i}\left(t\right)$, i=1,…,n, satisfy (A1’), one can obtain the following equation analog to (10):

(23) $$\hat{\phi }\left(t+1\right)=\left(I+\delta \left(t\right)\left\{\left[\Omega \left(t\right)+\Psi \left(t\right)\right]\left(\Gamma ⊗I_{2}\right)+\tilde{N}\left(t\right)\right\}\right)\hat{\phi }\left(t\right),$$

where $\Psi \left(t\right)=\mathrm{diag}\{\Psi_{1}\left(t\right),\ldots ,\Psi_{n}\left(t\right)\}$ and $\tilde{N}\left(t\right)=\left[{\tilde{N}}_{ij}\left(t\right)\right]$ with ${\tilde{N}}_{ij}\left(t\right)=-\sum_{k,k\neq i}\gamma_{ik}N_{ii}\left(t\right)$ for i=j and ${\tilde{N}}_{ij}\left(t\right)=\gamma_{ij}N_{ij}\left(t\right)$ for i≠j, i,j=1,…,n.

In an analogous way as in the previous section, instead of (11), the following equation is obtained for the mean of the corrected calibration parameters

(24) $$\bar{\phi }\left(t+1\right)=\left[I+\delta \left(t\right)\left(\bar{B}+\Sigma_{\eta }\right)\right]\bar{\phi }\left(t\right),$$

where $\bar{B}$ is as in (11) and $\Sigma_{\eta }=-\mathrm{diag}\left\{\frac{{\left(\sigma_{1}^{\eta }\right)}^{2}}{\alpha_{1}}\sum_{j}\gamma_{1j},0,\ldots ,\frac{{\left(\sigma_{n}^{\eta }\right)}^{2}}{\alpha_{n}}\sum_{j}\gamma_{nj},0\right\}$. Because of the additional term $\Sigma_{\eta }$, the sums of the rows of the matrix $\bar{B}+\Sigma_{\eta }$ are not equal to zero anymore, so that the convergence to consensus (as in Theorem 1) cannot be achieved in this case.

However, it can be seen from the structure of the recursion (24) that, if we assume that the measurement noise variances ${\left(\sigma_{i}^{\eta }\right)}^{2}$ are a priori known, we can use them to modify the basic algorithm (5) in the following way, ensuring again the asymptotic convergence to consensus:

(25) $$\begin{array}{c} {\hat{\theta }}_{i}\left(t+1\right)={\hat{\theta }}_{i}\left(t\right)+\delta \left(t\right)\left\{\sum_{j\in N_{i}}\gamma_{ij}\epsilon_{ij}^{\eta }\left(t\right)\left[\begin{array}{c} y_{i}^{\eta }\left(t\right)\\ 1 \end{array}\right]+\left[\begin{array}{cc} {\left(\sigma_{i}^{\eta }\right)}^{2}\sum_{j\in N_{i}}\gamma_{ij} & 0\\ 0 & 0 \end{array}\right]{\hat{\theta }}_{i}\left(t\right)\right\}, \end{array}$$

where $\epsilon_{ij}^{\eta }\left(t\right)={\hat{z}}_{j}^{\eta }\left(t\right)-{\hat{z}}_{i}^{\eta }\left(t\right)$ and ${\hat{z}}_{i}^{\eta }\left(t\right)={\hat{a}}_{i}\left(t\right)y_{i}^{\eta }\left(t\right)+{\hat{b}}_{i}\left(t\right)$, i=1,…,n.

The following theorem deals with the convergence of the above modification of the basic algorithm, when the measurement noise is present together with the communication errors. The convergence points will again depend on the measurement and communication noise realizations, in a similar way as in Theorem 3.

**Theorem** **4** ([17])**.** *Assume that the assumptions (A1’), (A2)–(A8) hold. Then, $\hat{\phi }\left(t\right)$, given by (25), converges to $i_{1}w_{1}+i_{2}w_{2}$ in the mean square sense and w.p.1, where $w_{1}$ and $w_{2}$ are scalar random variables satisfying $E\left\{w_{1}\right\}=\rho_{1}^{\prime }\hat{\phi }\left(0\right)$ and $E\left\{w_{2}\right\}=\rho_{2}^{\prime }\hat{\phi }\left(0\right)$.*

Notice that the above theorem was based on assumption (A2): indeed, when both {x(t)} and $\left\{\eta_{i}\left(t\right)\right\}$ are i.i.d. sequences, it is not surprising that the asymptotic consensus is achievable only provided $\sigma_{i}^{\eta }$, i=1,…,n, are known. However, we can replace the unrealistic assumption (A2) with (A2’) (introduced in Section 4 in the noiseless case) allowing correlated sequences {x(t)} which is almost always the case in practice. In such a way, the correlatedness problem present in the algorithm (24) can be overcame, without requiring any a priori information about the measurement noise process. The idea is to introduce instrumental variables in the basic algorithm in the way analogous to the one often used in the field system identification, e.g., [60,61]. Instrumental variables have the basic property of being correlated with the measured signal, and uncorrelated with noise. If $\left\{\zeta_{i}\left(t\right)\right\}$ is the instrumental variable sequence of the *i*-th agent, one has to ensure that $\zeta_{i}\left(t\right)$ is correlated with x(t) and uncorrelated with $\eta_{j}\left(t\right)$, j=1,…,n. Under A2’) a logical choice is to take the delayed sample of the measured signal as an instrumental variable, i.e., to take $\zeta_{i}\left(t\right)=y_{i}^{\eta }\left(t-d\right)$, where d≥1. Consequently, we present the following general calibration algorithm based on instrumental variables able to cope with measurement noise:

(26) $${\hat{\theta }}_{i}\left(t+1\right)={\hat{\theta }}_{i}\left(t\right)+\delta \left(t\right)\sum_{j\in N_{i}}\gamma_{ij}\epsilon_{ij}^{\eta }\left(t\right)\left[\begin{array}{c} y_{i}^{\eta }\left(t-d\right)\\ 1 \end{array}\right],$$

where d≥1 and $\epsilon_{ij}^{\eta }\left(t\right)={\hat{z}}_{j}^{\eta }\left(t\right)-{\hat{z}}_{i}^{\eta }\left(t\right)$, ${\hat{z}}_{i}^{\eta }\left(t\right)={\hat{a}}_{i}\left(t\right)y_{i}^{\eta }\left(t\right)+{\hat{b}}_{i}\left(t\right)$, i=1,…,n. Following the derivations from Section 3, one obtains from (26) the following relations involving explicitly x(t) and the noise terms:

(27) $$\begin{array}{c} {\hat{\phi }}_{i}\left(t+1\right)={\hat{\phi }}_{i}\left(t\right)+\delta \left(t\right)\sum_{j\in N_{i}}\gamma_{ij}\{\left(\Omega_{i}\left(t,d\right)+\Psi_{i}\left(t,d\right)\right)\left({\hat{\phi }}_{j}\left(t\right)-{\hat{\phi }}_{i}\left(t\right)\right)\\ +N_{ij}\left(t,d\right){\hat{\phi }}_{j}\left(t\right)-N_{ii}\left(t,d\right){\hat{\phi }}_{i}\left(t\right)\}, \end{array}$$

where

$$\Omega_{i}\left(t,d\right)=\left[\begin{array}{cc} \alpha_{i}\beta_{i}x\left(t\right)+\alpha_{i}^{2}x\left(t\right)x\left(t-d\right) & \alpha_{i}\beta_{i}+\alpha_{i}^{2}x\left(t-d\right)\\ \left(1+\beta_{i}^{2}\right)x\left(t\right)+\alpha_{i}\beta_{i}x\left(t\right)x\left(t-d\right) & 1+\beta_{i}^{2}+\alpha_{i}\beta_{i}x\left(t-d\right) \end{array}\right],$$

$$\Psi_{i}\left(t,d\right)=\eta_{i}\left(t-d\right)\left[\begin{array}{cc} \alpha_{i}x\left(t\right) & \alpha_{i}\\ \beta_{i}x\left(t\right) & \beta_{i} \end{array}\right],$$

$$N_{ij}\left(t,d\right)=\frac{\eta_{j}\left(t\right)}{\alpha_{j}}\left[\begin{array}{cc} \alpha_{i}y_{i}\left(t-d\right) & 0\\ \beta_{i}y_{i}\left(t-d\right) & 0 \end{array}\right]+\left[\begin{array}{cc} \frac{\eta_{j}\left(t\right)\eta_{i}\left(t-d\right)}{\alpha_{j}} & 0\\ 0 & 0 \end{array}\right]$$

and

$$N_{ii}\left(t,d\right)=\frac{\eta_{i}\left(t\right)}{\alpha_{i}}\left[\begin{array}{cc} \alpha_{i}y_{i}\left(t-d\right) & 0\\ \beta_{i}y_{i}\left(t-d\right) & 0 \end{array}\right]+\left[\begin{array}{cc} \frac{\eta_{i}\left(t\right)\eta_{i}\left(t-d\right)}{\alpha_{i}} & 0\\ 0 & 0 \end{array}\right].$$

In the same way as in (23), we have

(28) $$\hat{\phi }\left(t+1\right)=\left(I+\delta \left(t\right)\left\{\left[\Omega \left(t,d\right)+\Psi \left(t,d\right)\right]\left(\Gamma ⊗I_{2}\right)+\tilde{N}\left(t,d\right)\right\}\right)\hat{\phi }\left(t\right),$$

where $\Omega \left(t,d\right)=\mathrm{diag}\{\Omega_{1}\left(t,d\right),\ldots ,\Omega_{n}\left(t,d\right)\}$, $\Psi \left(t,d\right)=\mathrm{diag}\{\Psi_{1}\left(t,d\right),\ldots ,\Psi_{n}\left(t,d\right)\}$, $\tilde{N}\left(t,d\right)=\left[{\tilde{N}}_{ij}\left(t,d\right)\right]$, where ${\tilde{N}}_{ij}\left(t,d\right)=-\sum_{k,k\neq i}\gamma_{ik}N_{ii}\left(t,d\right)$ for i=j and ${\tilde{N}}_{ij}\left(t,d\right)=\gamma_{ij}N_{ij}\left(t,d\right)$ for i≠j, i,j=1,…,n.

To formulate a convergence theorem for (28), the following modification of (A4) is needed:

(A4’) $m\left(d\right)>{\bar{x}}^{2}$ for some $d=d_{0}\geq 1$.

This assumption implies that the correlation $m(d_{0})$ should be large enough. Similarly as in the case of (A4), it can be concluded that (A4’) implies that $-\bar{\Omega }\left(d\right)=-E\left\{\Omega_{i}\left(t,d\right)\right\}$ is Hurwitz. Similarly as in the above cases, let as introduce the similarity transformation

$$T^{\prime \prime }=\left[\begin{array}{ccc} i_{1} & i_{2} & T_{2n\times (2n-2)}^{\prime \prime } \end{array}\right],$$

where $T_{2n\times (2n-2)}^{\prime \prime }$ is an 2n×(2n−2) matrix, such that $\mathrm{span}\left\{T_{2n\times (2n-2)}^{\prime \prime }\right\}=\mathrm{span}\left\{\bar{B}{\left(d\right)}^{\prime \prime }\right\}$. Then, ${\left(T^{\prime \prime }\right)}^{-1}=\left[\begin{array}{c} \rho_{1}^{\prime \prime }\\ \rho_{2}^{\prime \prime }\\ S_{(2n-2)\times 2n}^{\prime \prime } \end{array}\right]$, where $\rho_{1}^{\prime \prime }$ and $\rho_{2}^{\prime \prime }$ are the left eigenvectors of $\bar{B}{\left(d\right)}^{\prime \prime }=E\left\{\Omega \left(t,d\right)\left(\Gamma \left(t\right)⊗I_{2}\right)\right\}=\bar{\Omega }\left(d\right)\left(\bar{\Gamma }⊗I_{2}\right)$ corresponding to the zero eigenvalue. The following theorem deals with the convergence of the instrumental variable algorithm (26). The convergence point, again, depends on the noise realization.

**Theorem** **5** ([16])**.** *Assume that the assumptions (A1’), (A2’), (A3), (A4’), (A5)–(A8) hold. Then $\hat{\phi }\left(t\right)$, given by (28) with $d=d_{0}$, converges to $i_{1}w_{1}+i_{2}w_{2}$ in the mean square sense and w.p.1, where $w_{1}$ and $w_{2}$ are scalar random variables satisfying $E\left\{w_{1}\right\}=\rho_{1}^{\prime \prime }\hat{\phi }\left(0\right)$ and $E\left\{w_{2}\right\}=\rho_{2}^{\prime \prime }\hat{\phi }\left(0\right)$.*

### 5.3. Asynchronous Broadcast Gossip Communication

So far we have shown how to deal with most of the practical challenges which emerge when dealing with real life SNs, such as communication dropouts, communication additive noise and measurement noise. However, in all of the above discussed algorithms we have implicitly assumed that all the nodes in the network share a common clock, based on which the recursions in (5), (25) or (26) can be implemented synchronously. Indeed, when introducing the basic algorithm we have assumed that the signal x(t) is being measured in discrete-time instances *t* by all the nodes. These instances are also used as time indexes of synchronous recursions of the above algorithms. Yet, there are many practical cases of SNs for which it is impossible or impractical to function synchronously. A typical example is the case when the nodes follow certain sleeping policies in order to minimize power consumption (e.g., [3]). For example, the nodes in SN shown in Figure 1, measuring air pollution or atmospheric conditions, may be programmed to make measurements less often during periods in which there is less traffic in the city. These types of situations are rigorously treated in the rest of this subsection.

Instead of the problem setup introduced in Section 3, assume now that the sensors are measuring a continuous-time signal x(t) at discrete points $t_{k}$, $t_{k}\in R^{+}$, k=1,2,…, $t_{k+1}>t_{k}$, producing the sensor outputs

(29) $$y_{i}\left(t_{k}\right)=\alpha_{i}x\left(t_{k}\right)+\beta_{i}+\eta_{i}\left(t_{k}\right),$$

where the $\alpha_{i}$ and $\beta_{i}$ are the same unknown parameters as in the previous subsections, and we also assume that the measurement noise $\eta_{i}\left(t_{k}\right)$, i=1,…,n, is present in the sensor readings.

Furthermore, since the goal is to remove dependence on a common global clock, it is now assumed that every node j∈N has its own local clock. For the sake of compact notation and simpler derivations, a single clock, called global virtual clock, is introduced, which ticks when any of the local clocks ticks. Hence, $t_{k}$ in (29) can be considered as the time in which the *k*-th tick of the virtual clock happend. To have a well defined situation, it is formally assumed that the ticks of the local clocks are independent, and that the intervals between any two consecutive ticks are finite w.p.1. It is also assumed, for the sake of simpler derivations, that the unconditional probability that the *j*-th clock ticked at an instance $t_{k}$ is $q_{j}>0$, independently of *k*. It is easy to verify that these conditions are satisfied for a typical model used in SNs, where it is assumed that the local clocks tick according to independent Poisson processes with rates $\mu_{j}$ (as in, e.g., [62,63]). This case will be adopted throughout this subsection. It directly follows that, in this case, the virtual global clock ticks according to a Poisson process with the rate $\sum_{j=1}^{n}\mu_{j}$.

According to the above assumptions, let us denote with $t_{l}^{j}$ the ticks of the local clock *j*, l=1,2,…. The communication protocol can then be defined in the following way. At each local clock tick, a node *j* makes the local sensor measurement, calculates the corrected sensor output $z_{j}\left(t_{l}^{j}\right)$ (based on the current estimates of calibration parameters $a_{j}$ and $b_{j}$), and broadcasts it to its out-neighbors $i\in N_{j}^{\mathrm{out}}$. We assume also that communication dropouts can happen, i.e., each node $i\in N_{j}^{\mathrm{out}}$ receives the transmitted message with probability $p_{ij}>0$. For the sake of clarity of presentation, we do not treat additive communication noise in this subsection. It is also assumed that the communication delay is negligible, so that, practically at the same time instant all the nodes which have received the broadcast, perform the local sensor reading, calculate their corrected outputs $z_{i}\left(t_{l}^{j}\right)$, and update the local estimates of their calibration parameters $a_{i}$ and $b_{i}$. This procedure is repeated for any local clock tick. The index of the node whose clock has ticked at instant $t_{k}$ is denoted by j(k), and let J(k) be the subset of the out-neighbors $i\in N_{j(k)}^{\mathrm{out}}$ which have received the broadcast message. Also, let $x\left(k\right)=x\left(t_{k}\right)=x\left(t_{l}^{j(k)}\right)$, $y_{i}\left(k\right)=y_{i}\left(t_{k}\right)=y_{i}\left(t_{l}^{j(k)}\right)$, $y_{j}\left(k\right)=y_{j}\left(t_{k}\right)=y_{j(k)}\left(t_{l}^{j(k)}\right)$, $z_{i}\left(k\right)=z_{i}\left(t_{k}\right)=z_{i}\left(t_{l}^{j(k)}\right)$, $z_{j}\left(k\right)=z_{j}\left(t_{k}\right)=z_{j(k)}\left(t_{l}^{j(k)}\right)$, $\eta_{i}\left(k\right)=\eta_{i}\left(t_{k}\right)=\eta_{i}\left(t_{l}^{j(k)}\right)$ and $\eta_{j}\left(k\right)=\eta_{j}\left(t_{k}\right)=\eta_{j(k)}\left(t_{l}^{j(k)}\right)$ for some *l*.

The measurement noise is treated as in the previous subsection, by using the delayed measurement $y_{i}\left(d_{i}\left(k\right)\right)$ as the instrumental variable

(30) $$\zeta_{i}\left(k\right)=y_{i}\left(d_{i}\left(k\right)\right),$$

where $d_{i}\left(k\right)$ is the global iteration number that corresponds to the closest past measurement of the node *i*. By using the same local criteria as in (3) and gradients as in (4), the following new recursion for updating the calibration parameters at node *i* is formulated:

(31) $${\hat{\theta }}_{i}\left(k\right)={\hat{\theta }}_{i}\left(k-1\right)+\delta_{i}\left(k\right)\gamma_{i,j(k)}\epsilon_{i,j(k)}\left(k\right)\left[\begin{array}{c} y_{i}\left(d_{i}\left(k\right)\right)\\ 1 \end{array}\right],$$

where:

- ${\hat{\theta }}_{i}\left(k\right)={\left[{\hat{a}}_{i}\left(k\right)\;\;{\hat{b}}_{i}\left(k\right)\right]}^{T}$,
- $\delta_{i}\left(k\right)$ is the step size given by $\delta_{i}\left(k\right)=\nu_{i}{\left(k\right)}^{-c}$, where $\nu_{i}\left(k\right)=\sum_{m=1}^{k}I\left\{i\in J\left(m\right)\right\}$ is the number of parameter updates of node *i* up to the iteration *k*, with 1/2<c≤1 (I{·} denotes the indicator function),
- $\epsilon_{i,j(k)}\left(k\right)={\hat{z}}_{j(k)}\left(k\right)-{\hat{z}}_{i}\left(k\right)$, where
  (32) $$\begin{array}{cc} {\hat{z}}_{j(k)}\left(k\right)= & {\hat{a}}_{j(k)}\left(k-1\right)y_{j(k)}\left(k\right)+{\hat{b}}_{j(k)}\left(k-1\right), \end{array}$$
  (33) $$\begin{array}{cc} {\hat{z}}_{i}\left(k\right)= & {\hat{a}}_{i}\left(k-1\right)y_{i}\left(k\right)+{\hat{b}}_{i}\left(k-1\right) \end{array}$$
  are the corrected outputs of node j(k) and node *i*.

The initial conditions are adopted to be ${\hat{\theta }}_{i}\left(0\right)={\left[1\;\;0\right]}^{T}$. Note that, according to the problem setup, at a given iteration *k* only the nodes i∈J(k) perform the above parameters update; for the rest of the nodes it holds that ${\hat{\theta }}_{i}\left(k\right)={\hat{\theta }}_{i}\left(k-1\right)$.

Computationally, the algorithm is as simple as the basic one, requiring only a few additions and multiplications in one iteration. Information needed at node *i* are: the local sensor measurement, the local instrumental variable, and the current output sent by an in-neighbor *j*. Knowledge of the global iteration index *k* (or $d_{i}\left(k\right)$) is not needed.

From the above definition of the step size $\delta_{i}\left(k\right)$ it can be concluded that it depends only on the number of local clock ticks, which makes the algorithm completely decentralized.

It should also be noticed that the instrumental variables in (31) can be selected in several ways. For example, instead of choosing (30), it can be practical to choose $\zeta_{i}\left(k\right)=y_{i}\left({\bar{t}}_{l}^{j,i}\right)$, where ${\bar{t}}_{l}^{j,i}$ is the time instant of a supplementary measurement of node *i*, just after the last step of the recursion (31) has been locally performed. This scheme is not assumed in the sequel, because of much more complicated notation; all the results can be easily transferred to this case.

Similarly as in the synchronous case, we introduce:

(34) $${\hat{\phi }}_{i}\left(k\right)=\left[\begin{array}{c} {\hat{g}}_{i}\left(k\right)\\ {\hat{f}}_{i}\left(k\right) \end{array}\right]=\left[\begin{array}{cc} \alpha_{i} & 0\\ \beta_{i} & 1 \end{array}\right]{\hat{\theta }}_{i}\left(k\right),$$

and

(35) $$\begin{array}{c} \epsilon_{i,j(k)}\left(k\right)=\left[\begin{array}{cc} cx(k) & 1 \end{array}\right]\left({\hat{\phi }}_{j(k)}\left(k\right)-{\hat{\phi }}_{i}\left(k\right)\right)+{\hat{a}}_{j(k)}\left(k\right)\eta_{j(k)}\left(k\right)-{\hat{a}}_{i}\left(k\right)\eta_{i}\left(k\right). \end{array}$$

Consequently, we have

(36) $$\begin{array}{cc} {\hat{\phi }}_{i}\left(k\right)= & {\hat{\phi }}_{i}\left(k-1\right)+\delta_{i}\left(k\right)\gamma_{i,j(k)}\{\left(\Omega_{i}\left(k\right)+\Psi_{i}\left(k\right)\right)\left({\hat{\phi }}_{j(k)}\left(k-1\right)-{\hat{\phi }}_{i}\left(k-1\right)\right)\\ & +N_{i,j(k)}\left(k\right){\hat{\phi }}_{j(k)}\left(k-1\right)-N_{ii}\left(k\right){\hat{\phi }}_{i}\left(k-1\right)\}, \end{array}$$

where

$$\Omega_{i}\left(k\right)=\left[\begin{array}{cc} \alpha_{i}\beta_{i}x\left(k\right)+\alpha_{i}^{2}x\left(k\right)x\left(d_{i}\left(k\right)\right) & \alpha_{i}\beta_{i}+\alpha_{i}^{2}x\left(d_{i}\left(k\right)\right)\\ \left(1+\beta_{i}^{2}\right)x\left(k\right)+\alpha_{i}\beta_{i}x\left(k\right)x\left(d_{i}\left(k\right)\right) & 1+\beta_{i}^{2}+\alpha_{i}\beta_{i}x\left(d_{i}\left(k\right)\right) \end{array}\right]$$

$$\Psi_{i}\left(k\right)=\eta_{i}\left(d_{i}\left(k\right)\right)\left[\begin{array}{cc} \alpha_{i}x\left(k\right) & \alpha_{i}\\ \beta_{i}x\left(k\right) & \beta_{i} \end{array}\right],$$

$$N_{i,j(k)}\left(k\right)=\frac{\eta_{j(k)}\left(k\right)}{\alpha_{j(k)}}\left[\begin{array}{cc} \alpha_{i}y_{i}^{0}\left(d_{i}\left(k\right)\right) & 0\\ \beta_{i}y_{i}^{0}\left(d_{i}\left(k\right)\right) & 0 \end{array}\right]+\left[\begin{array}{cc} \frac{\eta_{j(k)}\left(k\right)\eta_{i}\left(d_{i}\left(k\right)\right)}{\alpha_{j(k)}} & 0\\ 0 & 0 \end{array}\right]$$

and

$$N_{ii}\left(k\right)=\frac{\eta_{i}\left(k\right)}{\alpha_{i}}\left[\begin{array}{cc} \alpha_{i}y_{i}^{0}\left(d_{i}\left(k\right)\right) & 0\\ \beta_{i}y_{i}^{0}\left(d_{i}\left(k\right)\right) & 0 \end{array}\right]+\left[\begin{array}{cc} \frac{\eta_{i}\left(k\right)\eta_{i}\left(d_{i}\left(k\right)\right)}{\alpha_{i}} & 0\\ 0 & 0 \end{array}\right],$$

where $y_{i}^{0}\left(k\right)=\alpha_{i}x\left(k\right)+\beta_{i}$, with the initial conditions ${\hat{\phi }}_{i}\left(0\right)={\left[\alpha_{i}\;\;\beta_{i}\right]}^{T},i\in J(k)$.

Recursions (36) for i=1,…,n, can be written compactly as

(37) $$\hat{\phi }\left(k\right)=\left\{I+\left[\Omega \left(k\right)+\Psi \left(k\right)\right]\left(\Delta \left(k\right)\Gamma \left(k\right)⊗I_{2}\right)+\left(\Delta \left(k\right)⊗I_{2}\right)\tilde{N}\left(k\right)\right\}\hat{\phi }\left(k-1\right),$$

where:

- $\hat{\phi }\left(k\right)={\left[{\hat{\phi }}_{1}{\left(k\right)}^{T}\ldots {\hat{\phi }}_{n}{\left(k\right)}^{T}\right]}^{T},$
- $\Delta \left(k\right)=\mathrm{diag}\{\delta_{1}\left(k\right),\ldots ,\delta_{n}\left(k\right)\}$,
- $\Omega (k)=\mathrm{diag}\{\Omega_{1}\left(k\right),\ldots ,\Omega_{n}\left(k\right)\}$,
- $\Gamma \left(k\right)=[\Gamma {\left(k\right)}_{lm}]$, with $\Gamma {\left(k\right)}_{ll}=-\gamma_{l,j(k)}$ and $\Gamma {\left(k\right)}_{l,j(k)}=\gamma_{l,j(k)}$ for all l∈J(k), $\Gamma {\left(k\right)}_{lm}=0$, otherwise,
- $\Psi \left(k\right)=\mathrm{diag}\{\Psi_{1}\left(k\right),\ldots ,\Psi_{n}\left(k\right)\}$,
- $\tilde{N}\left(k\right)=\left[{\tilde{N}}_{lm}\left(k\right)\right]$, where ${\tilde{N}}_{ll}\left(k\right)=-\gamma_{l,j(k)}N_{ll}\left(k\right)$ and ${\tilde{N}}_{l,j(k)}\left(k\right)=\gamma_{l,j(k)}N_{l,j(k)}\left(k\right)$, for all l∈J(k), $\tilde{N}{\left(k\right)}_{lm}=0$, otherwise.

The initial condition is $\hat{\phi }\left(0\right)={\left[{\hat{\phi }}_{1}{\left(0\right)}^{T}\ldots {\hat{\phi }}_{n}{\left(0\right)}^{T}\right]}^{T}={\left[{\left[\alpha_{1}\;\beta_{1}\right]}^{T}\ldots {\left[\alpha_{n}\;\beta_{n}\right]}^{T}\right]}^{T}$.

Since we have formulated a slightly different problem setup than in Section 3, we introduce a new set of assumptions, and denote them using letter B:

(B1) {x(k)} is a stationary random sequence, bounded w.p.1, and satisfying the *ϕ*-mixing condition.

(B2) Let $\left\{t^{i,l}\right\}$, l=1,2,… represent time instants in which node *i* performs measurements. Then, $\min_{i}{\bar{r}}_{i}>m^{2}$, where m=E{x(k)} and ${\bar{r}}_{i}=E\left\{x\left(t^{i,l}\right)x\left(t^{i,l-1}\right)\right\}$, i=1,…,n.

(B3) Graph G has a spanning tree.

(B4) $\left\{\eta_{i}\left(k\right)\right\}$, i=1,…n, are zero-mean sequences of independent and bounded w.p.1 random variables. $\left\{\eta_{i}\left(k\right)\right\}$ is independent of the process {x(t)}, with $E\left\{\eta_{i}{\left(k\right)}^{2}\right\}={\left(\sigma_{i}^{\eta }\right)}^{2}$ for all *k*.

Assumptions (B3) and (B4) are essentially the same as (A3) and (A8).

The *ϕ*-mixing condition (B1) represents one of the strong mixing conditions, usually satisfied for sensory signals [64,65,66].

Assumption (B2) represents an extension of the assumption (A4), adapted to the presence of the instrumental variable $y_{i}\left(d_{i}\left(k\right)\right)$ in (31). It guarantees the persistence of excitation in the sense that the variance of x(k) must be greater than zero (for all *k*, because of stationarity) so that constant signals are not allowed [13,55]. However, it also ensures sufficent correlation between the instrumental variable and the current measurement, so that e.g., white noise signals are also not allowed. It can be easily derived [14] that (B2) is satisfied if the autocovariance function of x(t) is positive in a sufficiently large interval around zero. Also, if the rates $\mu_{j}$ are adjustable we can choose $\mu_{\min}=\min_{j\in N}\mu_{j}$ large enough, such that (B2) is always satisfied. Therefore, (B2) is, in general, not restrictive for processes having dominant low frequency spectrum, which is typical in practical applications of SNs.

Based on the above modified problem definition, the following result was proved in ref. [14], stating that both corrected gains and corrected offsets will converge to consensus points (which depend on the realizations of the stochastic processes) for all the nodes.

**Theorem** **6** ([14])**.** *Let Assumptions (B1)–(B4) be satisfied. Then $\hat{\phi }\left(k\right)$ given by (37) converges to ${\hat{\phi }}_{\infty }=\chi_{1}i_{1}+\chi_{2}i_{2}$ in the mean square sense and w.p.1, where $\chi_{1}$ and $\chi_{2}$ are random variables with bounded second moments.*

## 6. Discussion

### 6.1. Rate of Convergence

In the above subsections we did not discuss on how quickly the presented algorithms can achieve convergence to the specified points. Since the basic Equation (5) have constant step size, it can be concluded that the asymptotic convergence rate in the noiseless case is exponential. In the cases of measurement and additive communication noise, the convergence rate of the algorithms can be obtained following general methodology applied to the analysis of standard stochastic approximation algorithms. The following result gives an upper bound on the mean-square error with respect to the consensus point:

**Theorem** **7** ([17])**.** *Under the assumptions of any of the Theorems 3, 4 or 5, together with $\lim_{t\to \infty }\left(\delta {\left(t+1\right)}^{-1}-\delta {\left(t\right)}^{-1}\right)=d\geq 0$, there exists such a positive number $\sigma^{\prime }<1$ that for all $0<\sigma <\sigma^{\prime }$ the asymptotic consensus is achieved by the presented algorithms with the convergence rate $o(\delta {\left(t\right)}^{\sigma })$.*

It might be problematic to obtain the precise value of $\sigma^{\prime }$ in concrete applications. However, it can be shown that it directly depends on the sensor and network properties (encoded by matrix B(t) or B(t,d)) and on the connectivity of the underlying communication graph [67]. On one hand, if the number of nodes is increased without increasing network connectivity, the rate of convergence will decrease; however, if the graph connectivity is increased, the convergence rate will also increase. For example, if the graph is fully connected, the convergence rate will be high at the expense of very large number of communication links. In practice, a compromise between the rate of convergence and the network complexity needs to be found.

Another compromise to be found is between algorithm’s noise immunity and convergence rate. Indeed, according to [68], assuming that δ(t) is given as $\delta \left(t\right)=m_{1}/\left(m_{2}+t^{\mu }\right)$, $m_{1},m_{2}>0$, $\frac{1}{2}<\mu \leq 1$, the values of μ closer to $\frac{1}{2}$ give larger rate of convergence but higher sensitivity to noise; the values of μ closer to 1 give the opposite effect.

### 6.2. Stationarity of the Measured Signal

In the previous section, we made comments about all the introduced assumptions, explaining their practical applicability. Let us make some additional comments on the stationarity assumption for the random process {x(t)}, introduced in (A2), (A2’) and (B1). From the point of view of applications, it cannot be considered restrictive, since it encompasses a large variety of quickly and slowly varying real signals. This assumption is not essential for proving convergence of the presented algorithms: it has been introduced primarily for the sake of focusing on the essential structural aspects of the algorithm and avoiding complex notation [13,14,17]. Notice, according to Lemmas 2 and 3, that the similarity transformation *T* can be applied even in the case of time varying matrix $\bar{B}\left(t\right)$, owing to its specific structure; namely, we have $T^{-1}\bar{B}\left(t\right)T=\left[\begin{array}{cc} 0_{2\times 2} & 0_{2\times (2n-2)}\\ 0_{(2n-2)\times 2} & \bar{B}{\left(t\right)}^{*} \end{array}\right]$, where $\bar{B}{\left(t\right)}^{*}$ is Hurwitz and *T* is obtained from $\bar{B}\left(t\right)$ for any selected $t=t^{\prime }$. Moreover, notice that the conclusions of Theorem 1 hold, in general, provided the following unrestrictive condition holds: $\lim_{t}\prod_{\tau }\left(I-\bar{B}{\left(t-\tau \right)}^{*}\right)=0$. Also, it is possible to conclude directly that the results of the above theorems hold for changes of $\bar{B}{\left(t\right)}^{*}$ sufficiently slow. Moreover, it is not difficult to prove that the above convergence results exactly hold when the signal is asymptotically stationary.

### 6.3. Network Weights Design

As already discussed in the previous subsections, the implicit goal of the presented calibration scheme is to exploit the sensors with a priori good calibration properties by enforcing their dominating effect in the final consensus value to which all the nodes converge. This can be done in two ways, by adjusting the design weights $\gamma_{ij}$: (1) if the majority of sensors are “good”, we can set all $\gamma_{ij}$ for the neighborhood of any node *i* to the same value; or (2) if there is a smaller subset $N^{f}\subset N$ of a priori “good” sensors in the network we should appropriately tune the values of $\gamma_{ij}$. For this scenario, in this subsection, we give a more detailed analysis of the weights adjusting problem for the case of asynchronous communications treated in Section 5.3.

According to the theoretical results presented in detail in ref. [14], the dominant component of the random variables $\left[\chi_{1}\;\chi_{2}\right]$ in Theorem 6 is given by a weighted sum of the unknown sensor parameters $\alpha_{i}$ and $\beta_{i}$. The positive weights are determined by the left eigenvectors $w_{1}$ and $w_{2}$ of $\bar{B}$ corresponding to the zero eigenvalue. In turn, these weights are functions of the design parameters $\gamma_{ij}$, the wake-up probabilities $q_{j}$, and the dropout probabilities $p_{ji}$, i,j=1,…,n. Therefore, it is clear that the initial characteristics of a selected sensor *i* will have larger influence on the asymptotic consensus value if the appropriate elements of $w_{1}$ and $w_{2}$ are increased. This can be achieved in two ways:

1. By reducing the values of all the elements in the *i*-th row of $\bar{\Gamma }$, or
2. By increasing the values $\gamma_{ji}$, j≠i, from the *i*-th column (keeping in mind that $\bar{\Gamma }$ must be row stochastic).

Probabilities $q_{j}$ can in certain situations also be adjusted since they depend on the rate of the local clock of node *j*. By increasing the clock rate of the node *j*, the influence of that node on the asymptotic calibration parameter values achieved at consensus, is also increased. Adjusting dropout probabilities $1-p_{ji}$ might also be possible in certain situations: by decreasing the probability of a node *i* of receiving a message from a neighbor, we increase its influence on the asymptotic consensus. Hence, there are several design variables which can be adjusted so that the desired convergence point is achieved.

### 6.4. Macro Calibration for Networks with Reference Nodes

**Figure 2:**
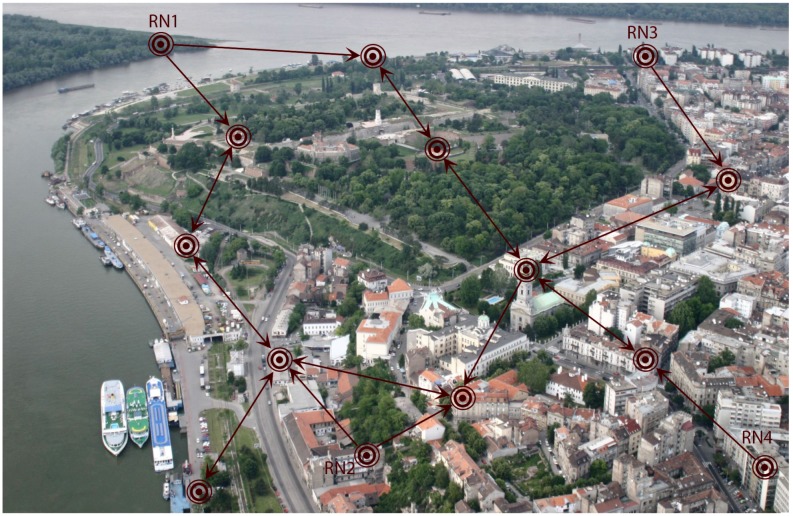
An example sensor network used in smart-city applications with multiple (four) reference nodes. The reference nodes (RNs) have fixed calibration parameters: only the rest of the nodes implement the given distributed sensor calibration recursions.

As discussed in the previous subsection, the selection of the weights in the matrix $\bar{\Gamma }$ is important for attaining the calibration goal of emphasizing a priori selected “good” sensors (“leaders”). Besides the described methods, this can be ultimately done by leaving the nodes from a set $N^{f}\subset N$ with unchanged calibration parameters (reference nodes), and only apply the recursions (31) (or (5), or (26)) to the rest of the nodes $i\in N-N^{f}$. An example of a SN with such topology corresponding to the smart city example in Figure 1 is shown in Figure 2.

In practice, this situation emerges, for example, when a SN needs to be expanded, i.e., when several uncalibrated sensors needs to be added to an already calibrated SN. In this subsection, the convergence results for this case are presented assuming asynchronous calibration algorithm [14].

First, we treat the special case in which $|N^{f}|=1$, i.e., there is only one reference sensor, and we want to calibrate the rest of the SN so that their calibrated outputs converge to the output of the reference sensor. For this case, all of the above results still hold, since the resulting communication graph will again have a spanning tree (with the reference node as a center node), which implies that (B3) (and (A3)) holds. Therefore, by applying the above convergence theorems, one concludes that the corrected gains and offsets ${\hat{\phi }}_{i}\left(k\right)$, i=1,…,n, will converge to the same value, dictated by the “leader”.

In the general case, assume, without loss of generality, that $N^{f}=\left\{1,2,\ldots ,n_{f}\right\}$, $n_{f}=\left|N^{f}\right|>0$, is the set of reference senors which have fixed parameters: $\phi_{i}^{f}=\left[\begin{array}{c} g_{i}^{f}\\ f_{i}^{f} \end{array}\right]$, $i\in N^{f}$. Assume that ${\bar{\phi }}^{f}=[\phi_{1}^{fT}\ldots \phi_{n_{f}}^{fT}{]}^{T}$. The calibration algorithms above are now applied in the same way, except that the reference nodes do not change the calibration parameters: ${\hat{\theta }}_{i}\left(k\right)={\hat{\theta }}_{i}\left(k-1\right)$ for all $i\in N^{f}$. Let $N-N^{f}=\{n_{f}+1,\ldots ,n\}$ and let ${\hat{\phi }}^{v}\left(k\right)=[{\hat{\phi }}_{n_{f}+1}{\left(k\right)}^{T}\ldots {\hat{\phi }}_{n}{\left(k\right)}^{T}{]}^{T}$ be the vector of all the calibration parameters to be tuned. In this case, all of the above theorems do not hold anymore, since, when $n_{f}>1$, the communication graph does not necessarily satisfy (B3) (the graph does not have a center node anymore, since two or more reference nodes are not mutually reachable). Hence, a separate convergence theorem is needed which treats this case:

**Theorem** **8** ([14])**.** *Let Assumptions (B1)–(B4) be satisfied and let all the nodes from $N-N^{f}$ be reachable from all the nodes in $N^{f}$. Then the algorithm (31), in which $\gamma_{ij}=0$ for all $i\in N^{f}$, provides convergence of ${\hat{\phi }}^{v}\left(k\right)$ in the mean square sense and w.p.1 to the limit defined by*

(38) $${\hat{\phi }}_{\infty }^{v}=-{\left({\bar{\Gamma }}^{v}⊗I_{2}\right)}^{-1}\left({\bar{\Gamma }}^{f,v}⊗I_{2}\right){\bar{\phi }}^{f},$$

*where matrices ${\bar{\Gamma }}^{v}$ and ${\bar{\Gamma }}^{f,v}$ are $\left(n-n_{f}\right)\times \left(n-n_{f}\right)$ and $\left(n-n_{f}\right)\times n_{f}$ submatrices of matrix $P^{-c}\bar{\Gamma }$, with indices $i,j=n_{f}+1,\ldots ,n$ and $i=n_{f}+1,\ldots ,n$, $j=1,\ldots ,n_{f}$, respectively; $P=\mathrm{diag}\{p_{1},\ldots ,p_{n}\}$ and c is defined in (31).*

It can be easily concluded that if the calibration parameters of all the reference nodes are the same, equal to some $\phi^{f}$, then the calibration parameters of the rest of the nodes will also converge to $\phi^{f}$. If this is not the case, it can be seen from (38) that the calibration parameters of different nodes will converge to different values. These values will be typically dictated by the reference sensors which are the closest to a given node.

### 6.5. Autonomous Gain Correction and Relationship with Time Synchronization

After presenting the most important aspects of the presented blind calibration methodology encompassing both noiseless and noisy environments, we will now make a few comments on the problem of its relationship with the algorithms for time synchronization in sensor networks, which has attracted a lot of attention (e.g., [40,41,42,43,44,45,46,47,69,70,71] and the references therein). Indeed, after coming back to the main measurement model, one easily realizes that in the case of time synchronization one has the form (1) with the absolute time *t* replacing the signal value x(t). The estimation schemes used in the mentioned analogous time synchronizaion algorithms are, indeed, related to the estimation of the parameters of the calibration functions (2) based on the use of local time measurements; however, they consist of one separate recursion for the relative drift estimation and one separate recursion for the estimation of offsets, relying on the obtained relative drifts. In ref. [72] this scheme was reformulated in the light of the calibration problem and the above described methodology. One starts from the difference model $\Delta y_{i}\left(t\right)=y_{i}\left(t+1\right)-y_{i}\left(t\right)=\alpha_{i}\Delta x\left(t\right)$, where Δx(t)=x(t+1)−x(t), and construct a gradient recursion for ${\hat{a}}_{i}$ following the above methodology, having in mind that $\Delta y_{i}\left(t\right)$ does not depend on $\beta_{i}$. On the other hand, the estimation of $b_{i}$ has to start from (1); it has the form of the recursion for ${\hat{b}}_{i}$ in (5), but should use ${\hat{a}}_{i}$ generated by first recursion. Such a combined gradient algorithm based on use of $\Delta y_{i}\left(t\right)$ resembles typical time synchronization algorithms. In general, it is important to observe that the introduction of *t* instead of x(t) in the basic relation (1) suffers from the very basic problem that unboundedness of the linear function contradicts the requirements for boundedness of the second order moments of x(t) (typical for stochastic approximation algorithms) and that it is not possible to guarantee convergence of the obtained recursions. This indicates that formal transfers of methodologies from one domain to the other should be done with extreme caution.

## 7. Simulation Results

In this section we present the results of extensive simulations, illustrating that the algorithms are applicable to real-world problems involving sensor networks with decentralized architectures. The results are presented using several figures which demonstrate all of the important properties theoretically addressed in the previous sections. In all of the simulations, a SN with ten nodes, with randomly generated communication graph satisfying assumption (A3), has been chosen. To show that the algorithms are applicable to a large variety of sensor characteristics, the sensor parameters $\alpha_{i}$ and $\beta_{i}$ have been generated randomly from uniform distribution with means one and zero, respectively, and with standard deviation 0.3.

**Figure 3:**
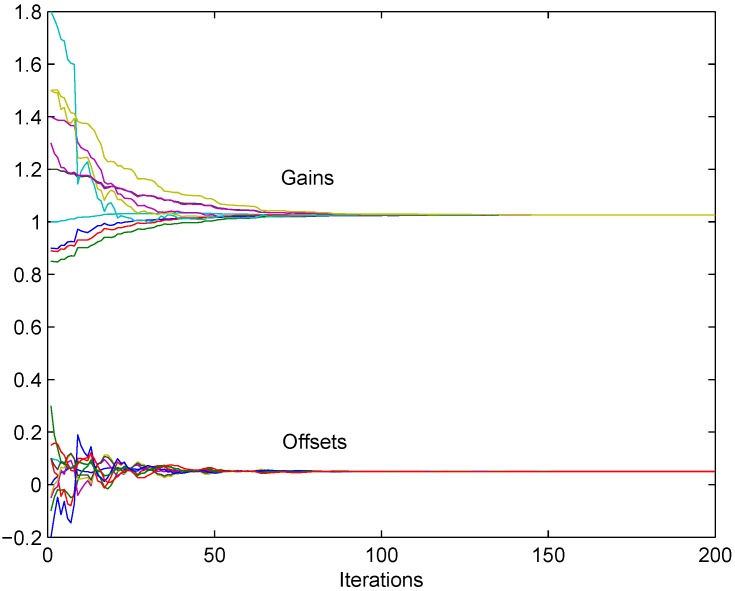
Noiseless synchronous algorithm without references: convergence to consensus is achieved for corrected gains and corrected offsets.

In Figure 3 the corrected gains ${\hat{g}}_{i}\left(t\right)$ and offsets ${\hat{f}}_{i}\left(t\right)$ obtained by the presented algorithm (5) in the noiseless case are presented, for a preselected step size, equal for all the nodes, δ=0.01. It is clear from the figure that the convergence to consensus, and, hence, implicit asymptotic calibration is achieved with the asymptotic values close the desired (one for corrected gains, and zero for corrected offsets).

**Figure 4:**
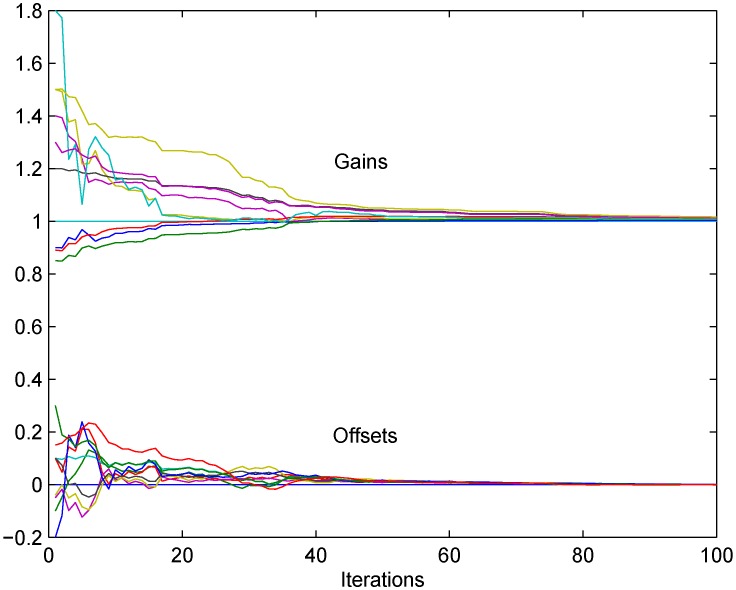
Noiseless synchronous algorithm with one reference sensor: convergence to the reference is chieved.

In Figure 4 the simulation results are presented for the situation in which the first node is set to be a reference node (“leader”) with perfect parameters $\alpha_{1}=1$ and $\beta_{1}=0$. Obviously, all of the rest of the nodes converge to this ideal characteristic.

**Figure 5:**
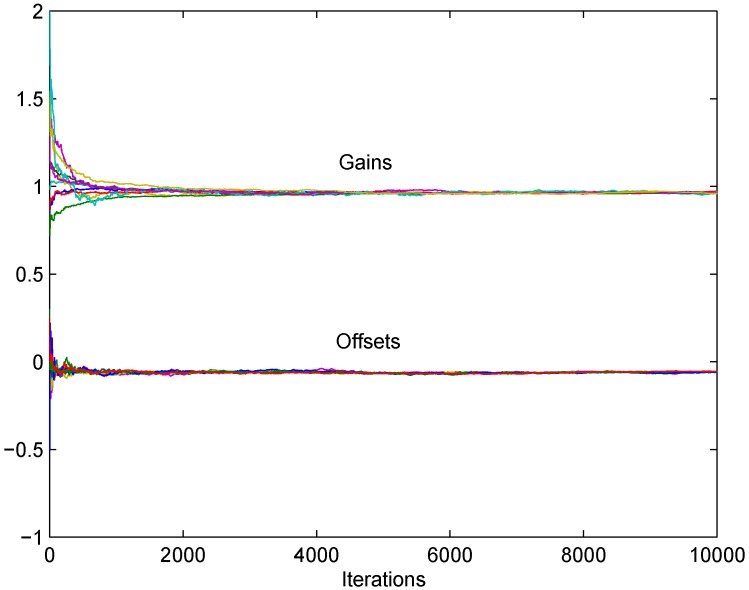
The modified algorithm (25): convergence to consensus is achieved for corrected gains and corrected offsets despite measurement noise presence.

In Figure 5 the corrected gains ${\hat{g}}_{i}\left(t\right)$ and offsets ${\hat{f}}_{i}\left(t\right)$ are depicted for the case in which all the theoretically discussed unreliabilities are included: communication dropouts with p=0.2 for all the links in the network, normally distributed communication additive noise with variance 0.1, and normally distributed measurement noises with variances different for all the nodes, uniformly generated in the range (0,0.1). In this case, in order to achieve convergence in the presence of noise, the time varying diminishing step-size sequences are chosen $\delta \left(t\right)=0.01/t^{0.6}$, equal for all the nodes. The algorithm which works in this case is the one based on the instrumental variables (26), so that the measured signal x(t) is assumed to be generated by a second order linear system with white noise at the input, resulting in a correlated sequence with zero mean and standard deviation one. It is obvious from the figure that the convergence is achieved despite the presence of all the introduced unreliabilities.

Next we simulate the asynchronous algorithm (31) which includes instrumental variables. It is assumed that the local clocks of all the nodes are driven by Poisson processes with the same rates.

**Figure 6:**
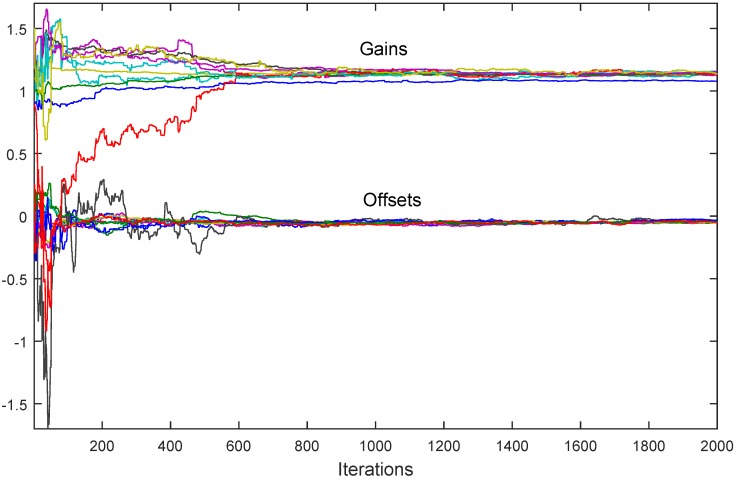
The asynchronous algorithm based on instrumental variables without reference sensors: convergence to consensus is achieved for corrected gains and corrected offsets.

In Figure 6, the corrected gains ${\hat{g}}_{i}\left(k\right)$ and offsets ${\hat{f}}_{i}\left(k\right)$ generated by (31) are depicted assuming the steps $\delta \left(k\right)=0.01/k^{0.6}$ and the presence of communication dropouts with probabilities $p_{ij}=0.2$ for each link. The measurement noises and the signal x(k) are assumed to be the same as in Figure 5.

**Figure 7:**
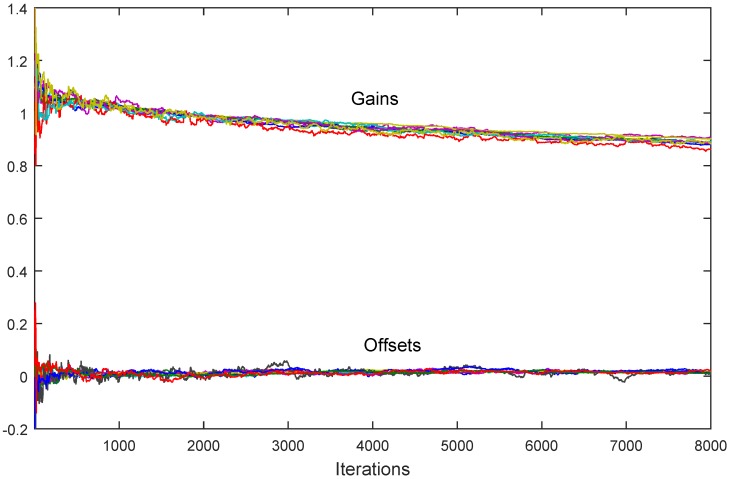
Stochastic gradient algorithm: convergence to consensus is not achieved.

In Figure 7 the necessity of introducing the instrumental variables is demonstrated when the noise is present. The basic algorithm (5), without instrumental variables, has been simulated and the convergence is not achieved in this case. It can be seen that all the corrected gains ${\hat{g}}_{i}\left(k\right)$ slowly converge to zero which is very undesirable.

**Figure 8:**
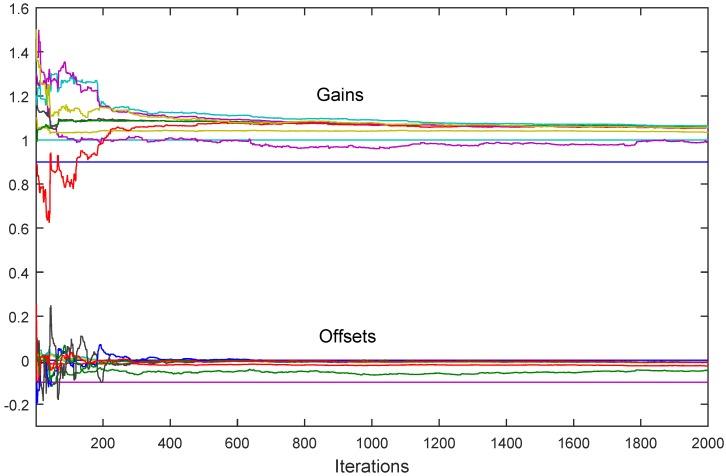
The asynchronous algorithm with two reference sensors with different characteristics: both the corrected gains and the corrected offsets converge to different values determined by (38).

Figure 8 illustrates the case discussed in Section 6.4 in which there are two reference sensors in the given SN. It can be seen from the figure that, as predicted by Theorem 8, the consensus is not achieved, and that all the calibration parameters converge to different values, determined by Equation (38).

## 8. Conclusions

In this paper, we consolidate the existing results on distributed recursive blind macro-calibration based on consensus, present the algorithms in a unified way, and provide additional analysis of several important theoretical and practical issues. The studied algorithms are completely decentralized, not requiring any fusion center. It was shown that the algorithms successfully perform on lossy sensor networks, characterized by unreliable communications, limited only to the local neighbors. Convergence properties have been presented under both noiseless conditions, and the conditions typical for noisy and unreliable environments. The practically important case of asynchronous communication based on a broadcast gossip scheme has also been treated. Extensive discussions have been provided, explaining in detail several important practical issues: convergence rate, design of tunable network weights, and calibration of sensor networks with multiple reference nodes. Extensive simulation results illustrating the behavior of the algorithms have been presented.

### Future Work

The presented results can be extended in several directions.

The first future direction, which is imposed naturally, is to extend the presented algorithms, or develop new ones, for the case when the nodes measure spatially varying but correlated signals. Treating this situation would drastically increase the practical applicability of the described methodology. The performance of such a scheme would highly depend on a priori knowledge about the interrelatedness of the measurements of different nodes.

Another direction of possible future work is to extend the results to include the possibility that individual nodes measure vector values (instead of scalars treated in this paper). This case would arise in practically frequent situations when there are multiple diverse sensors on each node, and when each sensor possibly measures different (overlapping) subsets of all the available sensed values. One possible idea for treating these situations is to use the wide existing literature in overlapping decentralized estimation (e.g., [20,73,74,75,76]). One typical example where this situation arises are networks of cameras with different view angles and scene coverage.

Third future direction emerges if one interprets the above results not in the context of calibration, but as pure synchronization (consensus) results for certain special types of linear time-varying systems. Indeed, the derivations and results of the presented convergence theorems open up a possibility of extending them to the case of synchronization of general higher order linear parameter-varying systems, which is a topic of high theoretical importance (e.g., [77] and references therein).

Finally, sensor networks often operate in hostile environments where battery usage and power consumption of the sensor nodes are of crucial importance. In this context it would be of high importance to analyze, for a given particular sensor network mission, how to formulate optimization problem and achieve optimal compromise between sensor network calibration and the nominal operation dictated by the given mission.
